# Structure and dynamics of polymyxin-resistance-associated response regulator PmrA in complex with promoter DNA

**DOI:** 10.1038/ncomms9838

**Published:** 2015-11-13

**Authors:** Yuan-Chao Lou, Tsai-Hsuan Weng, Yi-Chuan Li, Yi-Fen Kao, Wei-Feng Lin, Hwei-Ling Peng, Shan-Ho Chou, Chwan-Deng Hsiao, Chinpan Chen

**Affiliations:** 1 Institute of Biomedical Sciences, Academia Sinica, Taipei 115, Taiwan, ROC; 2 Institute of Molecular Biology, Academia Sinica, Taipei 115, Taiwan, ROC; 3 Department of Biological Science and Technology, National Chiao Tung University, Hsinchu 300, Taiwan, ROC; 4 Institute of Biochemistry, National Chung Hsing University, Taichung 40227, Taiwan, ROC; 5 Agricultural Biotechnology Center, National Chung Hsing University, Taichung 40227, Taiwan, ROC

## Abstract

PmrA, an OmpR/PhoB family response regulator, manages genes for antibiotic resistance. Phosphorylation of OmpR/PhoB response regulator induces the formation of a symmetric dimer in the N-terminal receiver domain (REC), promoting two C-terminal DNA-binding domains (DBDs) to recognize promoter DNA to elicit adaptive responses. Recently, determination of the KdpE–DNA complex structure revealed an REC–DBD interface in the upstream protomer that may be necessary for transcription activation. Here, we report the 3.2-Å-resolution crystal structure of the PmrA–DNA complex, which reveals a similar yet different REC–DBD interface. However, NMR studies show that in the DNA-bound state, two domains tumble separately and an REC–DBD interaction is transiently populated in solution. Reporter gene analyses of PmrA variants with altered interface residues suggest that the interface is not crucial for supporting gene expression. We propose that REC–DBD interdomain dynamics and the DBD–DBD interface help PmrA interact with RNA polymerase holoenzyme to activate downstream gene transcription.

## Abstract (web summary)

PmrA is a regulator of genes that are associated with antibiotic resistance. Here, the authors report the structure of the protein bound to DNA, and use NMR data to propose a mechanism for the regulation of gene transcription.

## Graphical abstract

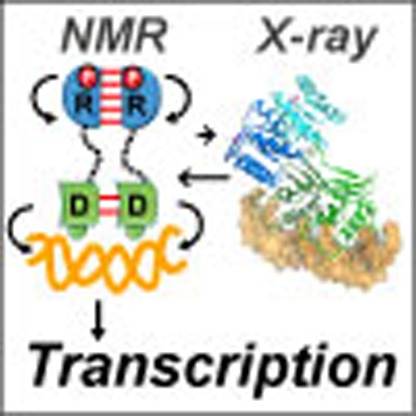

Two-component systems (TCSs) are adopted in bacteria, archaea, certain lower eukaryotes and higher plants to couple environmental stimuli with adaptive responses^1^. They are involved in a variety of processes, including virulence, antibiotic resistance and quorum sensing. TCSs are absent in mammals, so they are attractive targets for drug development^2^,^3^. The specific inhibitors of TCS systems are believed to work differently from conventional antibiotics and may be effective against various antibiotic-resistant bacteria^2^,^3^. Structural studies of TCSs that control virulence or antibiotic resistance, such as the PmrA/PmrB TCS^2^, are therefore crucial.

The PmrA/PmrB TCS is a major regulator of genes for lipopolysaccharide modification in the outer membrane of bacteria^4^. The response regulator PmrA, which belongs to the OmpR/PhoB family, functions as a transcription factor. The genes activated by PmrA, including *pbgP* and *ugd*, can encode enzymes to alter the composition of lipopolysaccharide, which increases the bacterial resistance to polymyxin B and other host-derived antimicrobial peptides^5^ or allows for bacterial survival within macrophages^6^.

A classical TCS typically consists of a transmembrane histidine kinase and a cytoplasmic response regulator protein^1^. The histidine kinase can sense stimuli and correspondingly regulate the signalling pathway. It autophosphorylates at a His residue, creating an active phosphoryl group that is transferred to the conserved Asp residue on the cognate response regulator protein to elicit adaptive responses, such as transcription activation. About 60% of response regulators function as transcription factors; they include the OmpR/PhoB family, which accounts for 30% of all TCSs^7^. Each OmpR/PhoB member contains an N-terminal receiver domain (REC) and a C-terminal DNA-binding domain (DBD), connected by a linker. Structural studies of inactive, unphosphorylated response regulators showed diverse interactions between REC and DBD. DrrB^8^, PrrA^9^ and MtrA^10^ have extensive interfaces between REC and DBD that inhibit phosphorylation of these response regulators by small-molecule phosphodonors^11^. The recognition helixes of PrrA^9^ and MtrA^10^ are occluded by the REC–DBD interactions, which prevents them from binding to DNA in the inactive state. However, there is no interaction between REC and DBD in the DrrD^12^ inactive structure^11^.

The common regulatory theme among OmpR/PhoB transcription factors is phosphorylation-induced dimerization for transcription activation. Phosphorylation of the OmpR/PhoB response regulator leads to the formation of a head-to-head REC dimer mediated by the α4–β5–α5 interface^7^. Dimerization of RECs brings two DBDs into close proximity for recognition of two tandem-repeat half-sites on the promoter. Several DBD–DNA complex structures revealed that DBDs bind to promoter DNA sequences in a head-to-tail manner^13^,^14^,^15^. It was also suggested that the flexible linker connecting the REC and DBD should allow the DBD to bind to DNA in any orientation, as dictated by the DNA sequence or protein–protein interaction specifically between DBDs^16^. However, the flexible linker may introduce high mobility of the complex and hamper crystal structure study of activated response regulators. In our previous study of RstA^14^, a response regulator with 16 residues in the linker region (Supplementary Fig. 1), we could determine only the crystal structures of a stand-alone REC dimer and the DBD–DNA complex. The crystallization of the full-length RstA–DNA complex was not successful.

Recently, Narayanan *et al.*^17^ presented the crystal structure of full-length KdpE in complex with its cognate DNA. For the active-like conformation of KdpE dimer in the complex, the two protomers are asymmetric with only the upstream protomer (the protomer bound to the upstream DNA) containing a substantial REC–DBD interface, which is stabilized by the interactions that involve seven residues and five water molecules. Structure–function studies show that the interface is necessary for transcription activation and may apply to other response regulators that act as transcription factors. However, sequence alignment of KdpE with other OmpR/PhoB response regulators (Supplementary Fig. 1) indicated that the seven residues involved in the interface are not conserved.

Here, we determine the crystal structure of BeF_3_^−^-activated PmrA in complex with the promoter DNA, which also reveals a REC–DBD interface in the upstream protomer, but the interactions of the interface are notably different from those observed in the KdpE–DNA complex structure. NMR studies further indicate that in solution, the RECs and DBDs of PmrA do not have close contacts but rather tumble separately in the absence or presence of DNA. Carr–Purcell–Meiboom–Gill (CPMG) relaxation dispersion experiments for the methyl groups of Leu, Val and Ile residues of PmrA suggest that a REC–DBD interface is transiently populated in the presence of DNA. Furthermore, β-galactosidase reporter assay of PmrA variants with altered interface residues concludes that the formation of a stable REC–DBD interface is not crucial for activating downstream gene transcription. From the model docking the PmrA–DNA complex structure with the *Escherichia coli* RNA polymerase σ^70^ holoenzyme (RNAPH)^18^, the REC–DBD interdomain dynamics and the DBD–DBD interface of PmrA may help PmrA search for the most suitable conformation for interacting with the RNA polymerase holoenzyme to activate downstream gene transcription. Our combined X-ray and NMR studies of the PmrA–DNA complex illustrate the significant differences between the crystal and solution states for multiple-domain proteins in bacterial two-component signal transduction.

## Results

### Double substitution of wild-type PmrA

To investigate the structural basis for promoter recognition by wild-type PmrA (WT-PmrA), protein samples should possess high stability and solubility. However, WT-PmrA aggregates severely during centrifugal concentration or when adding the phosphoryl analogue beryllofluoride (BeF_3_^−^) to activate protein samples. We screened different pH values, buffer types, salt concentrations and additives systematically but found no significant increase in solubility. We then calculated solvent-accessible surface areas from the X-ray structure of the REC domain^19^ and NMR structure of the DBD domain^20^ and identified two highly exposed hydrophobic residues, Trp^181^ and Ile^220^. The double-substitution W181G/I220D PmrA exhibited the best solubility and highest thermal stability (Supplementary Fig. 2a), which substantially improved NMR spectra quality as compared with WT-PmrA. The overlaid amide resonances in the ^1^H, ^15^N TROSY-HSQC spectra of the two protein molecules indicate that the double-substitution PmrA adopts a similar conformation as WT-PmrA (Supplementary Fig. 2b). For clarity, hereafter, we refer to the double-substitution W181G/I220D variant as PmrA.

### Overall structures of PmrA in complex with promoter DNA

PmrA was activated by the phosphoryl analogue BeF_3_^−^, which has been used to activate the REC domain to determine its activated structure^19^. The *pmra box* on the *pbgP* promoter of *Klebsiella pneumoniae* was verified previously^20^, and a series of various-length DNAs covering the half-1 and half-2 sites were mixed with an equal amount of BeF_3_^−^-activated PmrA for co-crystallization (Supplementary Table 1) to obtain the crystals of complexes with 25- and 26-bp DNA (Supplementary Fig. 3). We reveal the crystal structures of BeF_3_^−^-activated PmrA in complex with 25-bp DNA at 3.2 Å resolution and with 26-bp DNA at 3.8 Å resolution (Table 1). The space group of the PmrA–25-bp DNA crystal is C222, with two copies of the protein–DNA complex (complex-1 and complex-2) in the asymmetric unit (Supplementary Fig. 4a). The PmrA–26-bp DNA crystal has only one copy of the protein–DNA complex (complex-3; Supplementary Fig. 4b) packed in a different space group, P3_1_21. All the three complex structures contain a BeF_3_^−^-activated PmrA dimer (residues 1–219 modelled) binding to a double-stranded DNA (The upstream protomer that recognizes half-1 site is denoted PmrA-1 and the downstream protomer PmrA-2.) with similar conformations (Supplementary Fig. 4c). When superimposing all C_α_ coordinates of the PmrA dimer between the three complex structures, complex-2 exhibited the largest root mean square deviation (r.m.s.d.), 2.2 Å (Supplementary Table 2). In complex-2, PmrA-2 DBD interacts with the PmrA-1 REC in the symmetric unit, possibly causing the conformational deviation for complex-2. R.m.s.d. values between the three complex structures decreased to 0.6–1.0 Å with the PmrA-2 DBD excluded. Complex-1 structure, with the highest resolution and lowest r.m.s.d. as compared with the other two complex structures, was selected as the representative PmrA–DNA complex structure.

**Figure 1:**
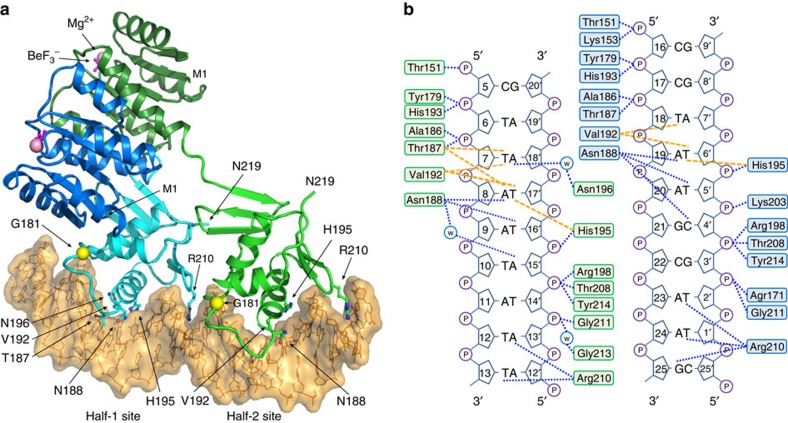
X-ray crystal structure of PmrA–DNA complex. (**a**) Cartoon presentation of the BeF_3_^−^-activated PmrA dimer in complex with the promoter DNA. The upstream protomer that recognizes half-1 site is denoted PmrA-1 and the downstream protomer PmrA-2. The REC and DBD of PmrA-1 are in blue and cyan and PmrA-2 in dark green and green, respectively. The BeF_3_^−^ and Mg^2+^ are shown as magenta sticks and pink spheres. The C_α_ atom of the mutated residue, Gly^181^, is a yellow sphere. The side chains that interact with bases are shown as sticks. (**b**) Schematic presentation of the detailed interaction between two PmrA molecules and the DNA. The H-bond and non-bonded contacts are indicated by blue and orange dotted lines, respectively.

In complex-1, the two RECs form a twofold symmetrical dimer, whereas the two DBDs bind to DNA in a head-to-tail orientation (Fig. 1a). The mutated residue, Gly^181^, is located in the N terminus of the transactivation loop and Asp^220^ is in the C-terminal end and not visible in the crystal structure. The interface between PmrA-1 and PmrA-2 is 1,336 Å^2^, with the two RECs contributing to 1,024 Å^2^. Each activated REC contains a five-stranded parallel β-sheet (β1–5) surrounded by five helices (α1–5) as well as a BeF_3_^−^ non-covalently bounded to Asp^51^ and a Mg^2+^ coordinated by BeF_3_^−^ and carbonyl oxygen from the main chains and side chains of surrounding residues (Supplementary Fig. 5a). Two activated RECs form a symmetric dimer mediated by the α4–β5–α5 interface, which is consistent with our previously determined crystal structure of the stand-alone REC dimer^19^ with an r.m.s.d. value of 0.55 Å for C_α_ atoms (Supplementary Fig. 5b).

The DBD contains a β-sheet (β6–8), a central three-helix core (α6–8) and a C-terminal β-hairpin (β9–10). In complex-1, the DBDs from PmrA-1 and PmrA-2 bind in tandem to DNA half-1 and half-2 sites. The contacts of PmrA-1 DBD with DNA span from nucleotide C5 (coding strand nucleotide) to A12′ (the prime sign indicates the template strand nucleotide), and PmrA-2 from C16 to T1′ (Fig. 1b). Basically, several residues spanning from the N terminus of helix α6 to the transactivation loop and of the C-terminal β-hairpin form H-bonds with a DNA phosphate backbone; Arg^210^ dips into the minor groove of the DNA to contact the bases; and Asn^188^ and Asn^196^, on the recognition helix α8, are inserted into the major grooves for base-specific recognition. The residues Thr^187^, Val^192^ and His^195^ are involved in hydrophobic interactions with bases.

In this complex, the DNA basically exhibits a B-form–like conformation with a mean base-pair helical rise and twist of 3.27 Å and 35.5°, respectively (3.32 Å and 36.0°, respectively, for B-DNA). Binding of the PmrA dimer bends the DNA gently around the protein with a curvature of ∼40° (Supplementary Fig. 6), similar to what was observed for the PhoB–DBD–DNA complex structure^13^. This curvature is due to narrowing of the minor groove width of sequences between the two half sites (T10 to C16), where the average minor groove width is narrowed to 9.7 Å (12 Å for B-DNA).

### DBD–DBD interactions

**Figure 2:**
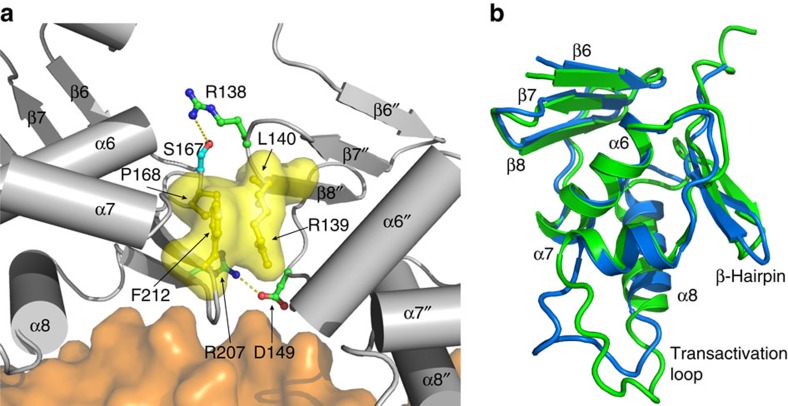
DBD–DBD interface and conformational changes in DBD. (**a**) The interactions between two DBDs bound with the promoter DNA. The DBD in PmrA-1 is on the left and in PmrA-2 is on the right. The side chains that form salt bridge interactions are shown with sticks and those that form hydrophobic contacts are yellow sticks and surfaces. (**b**) Comparison of structures of isolated PmrA DBD (green) and the DBD in PmrA-1 (blue). The r.m.s.d. value between two structures is 1.02 Å for C_α_ atoms from residues 127–178 (β6 to α7), which suggests that the DNA binding and DBD–DBD interaction altered the conformations of the transactivation loop, the recognition helix α8 and the C-terminal β-hairpin.

The two DBDs fit complementarily to each other with an interface of 291.8 Å^2^ in complex-1. The C-terminal β-hairpin and the loop between α6 and α7 of the PmrA-1 DBD interacts with the β7–β8 loop of the PmrA-2 DBD (Fig. 2a). The side chains of Arg^207^ (PmrA-1) and Asp^149^ (PmrA-2) establish a salt bridge, and those of Ser^167^ (PmrA-1) and Arg^138^ (PmrA-2) form an H-bond. Also, Pro^168^ and Phe^212^ from PmrA-1 as well as Arg^139^ and Leu^140^ from PmrA-2 constitute a hydrophobic cluster. Complex-2 and complex-3 feature similar interactions between the two DBDs, with interfaces of 303.7 and 284.1 Å^2^, respectively. We compared the structures of DBD in the free and DNA-bound states. The r.m.s.d. value between PmrA-1 DBD in complex-1 and the free stand-alone DBD NMR structure is large, with 2.26 Å for Cα atoms from residues 127–216. However, the r.m.s.d. value decreases to 1.02 Å if only residues 127–178 (the N-terminal β-sheet to helix α7) are superimposed (Fig. 2b). The conformations of the transactivation loop, the recognition helix α8 and the C-terminal β-hairpin are altered when DBD binds to DNA.

### REC–DBD interface in PmrA-1

**Figure 3:**
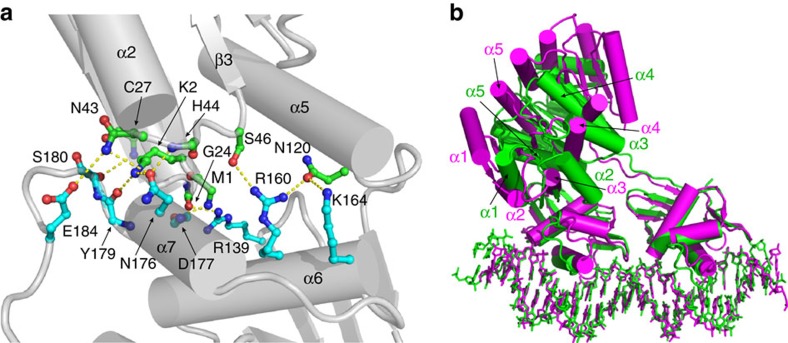
The REC–DBD interface and structure comparison of PmrA and KdpE complex structures. (**a**) The REC–DBD interface in PmrA-1. Extensive H-bond contacts, connected with yellow dotted lines, identified between residues from the N terminus, α1–β2 loop, α2–β3 loop, α5 of REC and those from α6, α7 and the transactivation loop of DBD, establishing an interacting interface of 702 Å^2^. (**b**) Structural superimposition of DBD domains in KdpE (magenta) and PmrA (green) complex structures. The conformations of DNA and DBD domains are similar in the two proteins, but the REC–DBD interfaces differ, for distinct orientations of the REC dimer.

In complex-1, the two RECs form a head-to-head dimer with the two DBDs bound to DNA in a head-to-tail orientation. We wondered what conformation the two linkers adopt to connect the symmetric RECs to the asymmetric DBDs. In PmrA-2, the REC and DBD are linked by an extended linker without any interdomain interaction. Interestingly, in PmrA-1, the linker forms a turn-like conformation stabilized by an H-bond and a salt bridge interaction (Supplementary Fig. 7), and REC and DBD exhibit extensive contacts via an interface of 702 Å^2^, with 11 H-bond interactions identified between residues from the N terminus, α1–β2 loop, α2–β3 loop, α5 of REC and side chains from α6, α7, and transactivation loop of DBD (Fig. 3a). The interface interactions identified in the three complexes are also similar (Supplementary Fig. 8 and Supplementary Table 3). Importantly, this interface differs from that in the KdpE–DNA complex structure^17^, yet the interface in KdpE is smaller (375 Å^2^) and the interactions involve different residues from the β3, α3−β4 loop of REC, five water molecules and side chains from the α6, α7 of DBD. Structural alignment of the KdpE DBD to the DBD of PmrA shows that the differences in REC–DBD interfaces lead to distinct orientations of the REC dimers (Fig. 3b). Sequence alignment of PmrA, KdpE and other OmpR/PhoB members shows that the interface residues in PmrA and KdpE are not conserved (Supplementary Fig. 1).

However, our previous NMR study showed that REC amide resonances from BeF_3_^−^-activated PmrA in complex with DNA are similar to those from the stand-alone BeF_3_^−^-activated REC dimer^20^, which suggests that the inclusion of DBD and addition of DNA do not greatly change the chemical environment of the PmrA REC dimer. Therefore, the preliminary NMR findings do not agree with the extensive REC–DBD interactions identified in PmrA-1 in the crystal structure. A thorough NMR investigation is necessary.

### NMR assignments on PmrA with or without DNA

**Figure 4:**
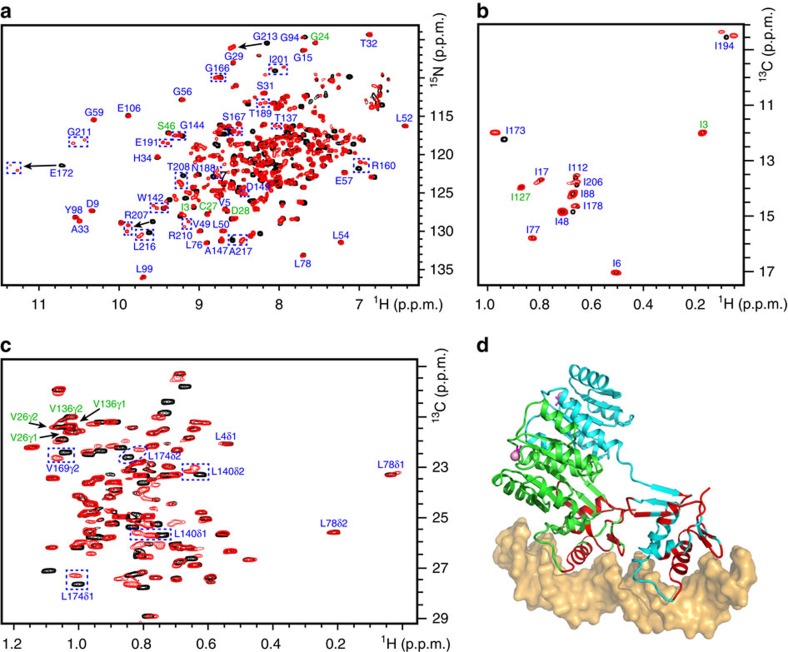
NMR investigations of the PmrA–DNA complex. The overlaid two-dimensional (2D) ^1^H, ^15^N TROSY-HSQC spectra (**a**) and 2D ^1^H, ^13^C HSQC spectra, showing the methyl resonances of Ile (**b**), Leu and Val (**c**), acquired from PmrA in the absence (black) and presence of DNA (red). The residues that show two sets of amide or methyl resonances in the DNA-bound state are boxed with blue dotted rectangles or ellipses and those involved in the REC–DBD interface are labelled in green. (**d**) All the residues with two resonance peaks are mapped on the structure (in red), showing that they are mainly located within the DBD–DBD and DBD–DNA interfaces.

We successfully prepared the methyl-protonated {Ile(δ1 ^13^CH_3_), Leu(^13^CH_3_, ^12^CD_3_), Val(^13^CH_3_, ^12^CD_3_)} U-[^2^H, ^13^C, ^15^N] sample of PmrA at pH 8.0 and identified 193 of 217 backbone amide resonances (with six Pro residues excluded; Fig. 4a). The 24 missing peaks are mainly located in the flexible regions, such as the N terminus (residues 1–2), the linker region (residues 117–125) and the transactivation loop of DBD (residues 181, 183, 187 and 189–192). The assignment of methyl resonances involved the ‘out-and-back' methyl-detected 3D HMCM(CG)CBCA experiment^21^, with 13/13 (100%) Ile δ1 methyl resonances, 69/72 (96%) Leu methyl resonances and 24/24 (100%) Val methyl resonances clearly identified (Fig. 4b,c). In BeF_3_^−^-activated PmrA, all amide and methyl resonances exhibit only one set of resonance peaks, and the REC and DBD domains do not closely interact with each other because the amide peaks from the stand-alone BeF_3_^−^-activated REC dimer and stand-alone DBD domain superimpose well with the peaks from BeF_3_^−^-activated PmrA (Supplementary Fig. 9).

The backbone amide resonance assignment of BeF_3_^−^-activated PmrA in complex with DNA involved use of TROSY-HNCA and HNN-NOESY^22^. In the DNA-bound state, the amides in the N terminus (residues 1–2) and the linker region (residues 117–125) are still missing, but several in the transactivation loop can be assigned because of close interactions with DNA (Fig. 4a). Finally, 19 missing amide resonances are not assigned. Similarly, with the methyl-detected experiment, we could assign most of the methyl groups in BeF_3_^−^-activated PmrA with DNA, except those from residues Leu^190^, Val^192^ and Ile^201^, which disappear after the DNA addition (Fig. 4b,c).

**Figure 5:**
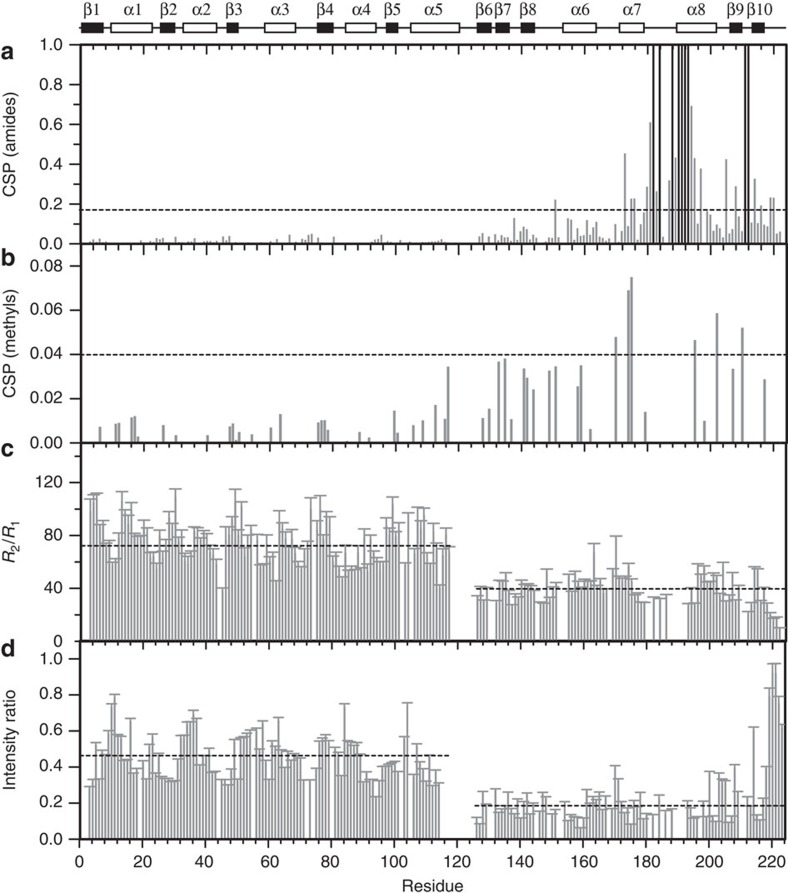
Weighted chemical shift perturbations (CSPs) and NMR dynamics on PmrA with and without DNA. CSP values for backbone amide resonances (**a**) and for methyl resonances (**b**). The black bars represent the residues that can be identified in the DNA-bound state but are missing without DNA. The dotted lines indicate the mean Δ*δ* value plus 1 s.d. of Δ*δ* values. (**c**) The *R*_1_ and *R*_2_ values of BeF_3_^−^-activated PmrA are measured in duplicate and the *R*_2_/*R*_1_ values are calculated. The average *R*_2_/*R*_1_ values are 72.5 and 40.1 for residues in REC and DBD, respectively, and are marked by dotted lines. Error bars represent fitting errors. (**d**) The intensity ratios of amide resonances in the DNA-bound state to free state. Error bars are noise-to-signal ratios. The mean intensity ratios are 0.46±0.10 for REC and 0.19±0.09 for DBD as indicated by the dotted lines.

In the DNA-bound state, many amides and methyl groups in DBD show two sets of resonance peaks, including Asn^188^, Thr^189^, Glu^191^, Ile^194^, Arg^210^ and Gly^211^, which bind to different DNA sequences in the crystal structure, and Thr^137^, Leu^140^, Trp^142^, Gly^144^, Asp^149^, Gly^166^, Arg^207^ and Thr^208^, which are located in the DBD–DBD interface (Fig. 4a–c). We mapped all of the residues with two resonance peaks on the structure, showing that these residues are mainly located within the DBD–DBD and DBD–DNA interfaces (Fig. 4d). Calculation of the weighted chemical shift perturbations (CSPs) of the amide and methyl resonances with and without DNA (Fig. 5a,b) shows that the residues with significant CSPs (Δ*δ*>Δ*δ*_average_+s.d.) are also mostly located within the DBD–DBD and DBD–DNA interfaces. In the PmrA–DNA complex structure, the two DBDs bind to the half-1 and half-2 sites to constitute a head-to-tail interface. Therefore, the asymmetric DBD–DBD and DBD–DNA interactions between two PmrA protomers in complex with DNA are also observed on NMR.

However, the REC residues, such as Ile^3^, Gly^24^, Val^26^, Cys^27^, Asp^28^, Ser^46^, Ile^127^ and Val^136^, which are located in the REC–DBD interface, exhibit only one set of NMR resonances in the DNA-bound state (Fig. 4a–c). None of these residues show significant CSPs (Fig. 5a,b). The REC–DBD interface identified in PmrA-1 in the complex crystal structure may be too transient to reveal a second set of NMR resonances or to cause a detectable CSP. Furthermore, the amide resonances of linker residues are missing with and without DNA, which implies that they do not form a stable conformation in either state. Characterizing the dynamic properties of the REC and DBD domains would be of interest.

### RECs and DBDs tumble separately in solution

To further understand the motions of REC and DBD in the BeF_3_^−^-activated PmrA, we performed NMR backbone dynamics study. The amide transverse (*R*_2_) and longitudinal (*R*_1_) relaxation rates are sensitive to the overall tumbling rate, which is modulated by molecule weight. A molecule with higher molecular weight exhibits a higher *R*_2_ rate and a longer overall rotational correlation time. Measurements of the BeF_3_^−^-activated PmrA show that the average *R*_1_ values are slightly different for residues in both domains, but the average *R*_2_ rates are rather distinct, with values of 48.9 and 34.2 s^−1^ for residues of the REC and DBD, respectively (Supplementary Fig. 10). The resulting mean *R*_2_/*R*_1_ values are 72.5 and 40.1 for the REC and DBD (Fig. 5c), which correspond to an overall rotational correlation time of 20 and 14 ns, respectively. Therefore, the REC and DBD tumble with different rotational correlation times in solution. This finding reflects that in BeF_3_^−^-activated PmrA, the two RECs form a stable dimer and connect to the two DBDs by flexible linkers without any close contact so that these domains can rotate separately in solution.

In the PmrA–DNA complex state, accurate measurement of the *R*_2_ relaxation rate of PmrA becomes difficult because of the large molecular weight of the resulting complex (∼70 kDa). Instead, we used a simplistic approach in measuring only two TROSY spectra, for the DNA-bound state and the free state, to calculate the intensity ratios of amide resonances (Fig. 5d). To measure the intensity of amide resonances more accurately, we increased the repetition delay to 5 s for the full recovery of longitudinal magnetization^23^. The resulting intensity ratios of amide resonances showed that the REC dimer and the two DNA-binding DBDs exhibit distinct average intensity ratios of 0.46±0.10 for REC and 0.19±0.09 for DBD (excluding the C-terminal seven residues), respectively. These results suggest that the mean enhanced *R*_2_ rate is smaller for REC than DBD and the apparent molecular weight is smaller for REC than DBD. Indeed, in the PmrA–DNA complex, the two DBDs are tightly bound to DNA and to each other, thereby constituting a complex with higher apparent molecular weight than the REC dimer. The intensity ratios of amide resonances in the DNA-bound to free state illustrate that for BeF_3_^−^-activated PmrA in complex with DNA, REC and DBD are connected by a flexible linker and the REC dimer and two DBDs with DNA tumble separately in solution.

### Slow dynamics for the methyls in the REC–DBD interface

**Figure 6:**
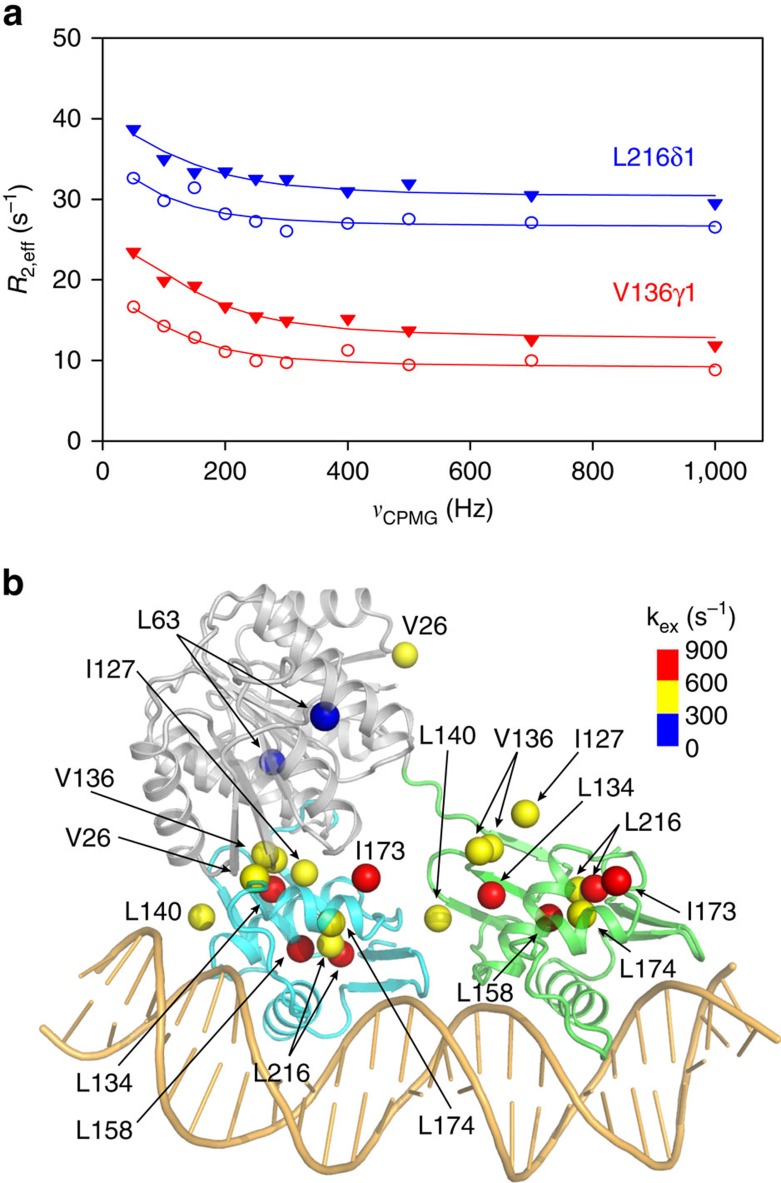
Slow dynamics probed by methyl-TROSY Carr–Purcell–Meiboom–Gill (CPMG) relaxation dispersion. (**a**) The dispersion data measured at 600 MHz (open circles) and 850 MHz (filled triangles) are shown for methyl groups of Val^136^ (red) and Leu^216^ (blue). Lines show individual fits to a two-site exchange process. (**b**) The methyl groups that exhibit slow dynamics detected by relaxation dispersion data are indicated by coloured spheres, to show the amount of exchange rate constants (*k*_ex_), derived from successful fitting of relaxation dispersion profiles.

To better understand the slow motions (micro-to-millisecond timescale) on PmrA in the presence of DNA, we performed a series of NMR relaxation dispersion experiments, which are capable of probing invisible, low-populated conformations in protein folding, ligand binding and allosteric regulation^24^,^25^,^26^. Because of limited solubility and high molecular weight of the PmrA–DNA complex, we used methyl-TROSY relaxation dispersion experiments^27^ to probe the slow dynamics in this complex. Methyl groups experiencing slow timescale motions show changes in effective relaxation rate, *R*_2,eff_, measured as a function of the frequency of refocusing pulses *ν*_CPMG_ (Fig. 6a and Supplementary Fig. 11). Measurements at both 600 and 850 MHz were fitted individually by use of the software relax^28^ to a two-site exchange process (A↔B), yielding populations (p_A_ and p_B_), exchange rate constants (*k*_ex_=*k*_AB_+*k*_BA_) and the ^1^H and ^13^C chemical shift differences between the two states^27^ (Supplementary Table 4). In total, 12 PmrA methyl groups exhibit slow dynamics on binding to DNA. These methyl groups are basically clustered in two regions (Fig. 6b): the core of DBD (Leu^158^, Leu^174^ and Leu^216^) and the REC–DBD interface in PmrA-1 (Val^26^, Ile^127^, Leu^134^, Val^136^ and Leu^140^). The relaxation dispersion profiles of these interface methyl groups further fitted globally reveal a single exchange process with p_A_ and p_B_ populations of 11% and 89% (±8%), respectively, and *k*_ex_=560±49 s^−1^. This result demonstrates that a transient formation of a REC–DBD interface is detected in solution for BeF_3_^−^-activated PmrA in complex with DNA.

We also measured the methyl-TROSY relaxation dispersion of BeF_3_^−^-activated PmrA in the absence of DNA at both 600 and 850 MHz. The fitting of all dispersion profiles to a two-site exchange process shows that only the methyl groups of three residues, Leu^40^, Leu^143^ and Ile^194^, exhibit slow dynamics (Supplementary Fig. 11 and Supplementary Table 4). However, they are not located in the REC–DBD interface. The absence of interface residues exhibiting slow dynamics indicates lack of the transiently populated state observed in the PmrA–DNA complex. DNA binding appears to be necessary for the transient formation of an REC–DBD interface in the upstream protomer of the PmrA dimer.

### Promoter recognition and transcription activation

**Figure 7:**
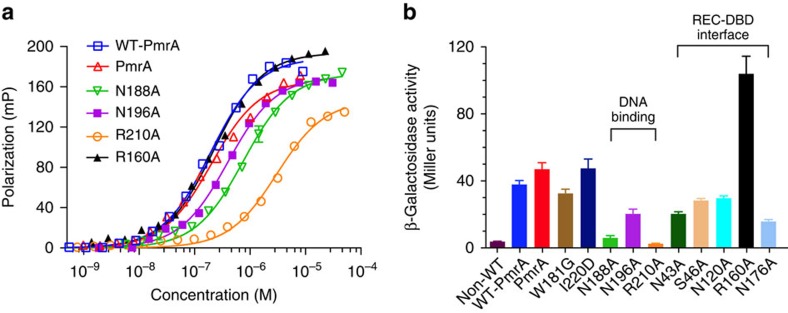
Promoter recognition and transcription activity. (**a**) The bindings between PmrA, WT-PmrA or its variants and DNA measured by fluorescence polarization experiments are fitted by a one-site binding model. See also Supplementary Fig. 11 for binding affinities. (**b**) β-Galactosidase reporter assay in *K. pneumoniae* carrying the plasmid that expresses PmrA, WT-PmrA or its variants and the reporter plasmid that contains the *pbgP* promoter in front of the *lacZ* gene. The expression of PmrA, WT-PmrA or its variants was induced by isopropyl-β-D-thiogalactoside (IPTG, 1 mM ml^−1^) until the cells grew to the mid-logarithmic phase. The β-galactosidase assay in cells that carry WT-PmrA plasmid but without the addition of IPTG is denoted non-WT, which represents the production of β-galactosidase induced by endogenous PmrA. Reporter assay results are expressed as Miller Units. All experiments were performed in triplicate. Error bars are defined as s.d.

To establish whether PmrA is biologically active and the formation of extensive REC–DBD interface is important for promoter recognition and transcription activation, we performed *in vitro* fluorescence polarization experiments (Fig. 7a and Supplementary Fig. 12) to measure the binding affinities between PmrA, WT-PmrA or its variants, and fluorescence-labelled DNA as well as *in vivo* β-galactosidase reporter assay (Fig. 7b) to monitor the transcription activity of all protein constructs. PmrA (the double-substitution W181G/I220D variant) retains full activity in promotor recognition (*K*_d_ values for WT-PmrA and PmrA to DNA of 193.5±7.7 and 200.6±8.2 nM, respectively) and transcription activation as for WT-PmrA. The residue Trp^181^ is in the N terminus of the transactivation loop, and the single-mutant W181G exhibits slightly lower transcript activity than that of WT-PmrA. The replacement of residues involved in DNA recognition by alanine reduces the DNA-binding affinity significantly (*K*_d_ values for N188A, N196A and R210A to DNA of 762.2±12.3, 398.9±10.9 and 3036.8±11.7 nM, respectively) and hence abolishes the activity in transcription. The mutants that change residues in the REC–DBD interface mostly exhibit a comparable DNA-binding affinity to that of WT-PmrA (Supplementary Fig. 12), with the exception of N176A (*K*_d_=364.9±11.6 nM), which therefore reveals decreased activity in transcription. Interestingly, induced expression of R160A leads to a significant increase (∼2.7-fold) in transcription, but the production of N43A, S46A and N120A results in slight-to-moderately reduced transcription. Western blot analysis (Supplementary Fig. 13) shows that the expression of most of the protein constructs in *K. pneumoniae* are equivalent to that of WT-PmrA, but N43A and S46A show decreased expression, which suggests that the β-galactosidase activity of both mutants can be higher if their expression levels resemble that of WT-PmrA. The alterations in interface residues mostly do not significantly interfere with their activities in promoter recognition and transcription activation. These analyses show that PmrA is biologically active and the formation of an REC–DBD interface is not crucial for activating downstream gene transcription.

## Discussion

The details of activated full-length OmpR/PhoB response regulators bound to DNA were lacking until the KdpE–DNA complex structure was revealed^17^. In this study, we present the PmrA–DNA complex structure. However, the intra-molecular REC–DBD interactions greatly differ between the two structures. Sequence alignment shows that the interface residues in two proteins are not conserved (Supplementary Fig. 1). We also found that the lengths of linkers between the REC and DBD differ among the OmpR/PhoB members. The linkers of DrrB, DrrD, PrrA and MtrA, whose inactive full-length crystal structures have all been determined^8^,^9^,^10^,^11^,^12^, and of KdpE and PmrA are shorter than those of other response regulators, such as RstA and OmpR. Longer linkers likely introduce greater mobility to the full-length response regulators and hamper their crystallization. The poor conservation in the interface residues and the variation in lengths of linkers suggest that the REC–DBD interfaces identified in activated PmrA and KdpE are unique and may not be conserved in other OmpR/PhoB members.

The most interesting finding is the discrepancy between the X-ray structure and NMR findings. In the PmrA-1 of the PmrA–DNA complex structure, the REC establishes extensive contacts with DBD. Similar crystal structures are obtained from two crystals of distinct shapes and space groups, growing with different lengths of DNA in different conditions, which rules out the possibility of a crystal packing effect. However, NMR assignment and dynamics study suggest that RECs and DBDs in BeF_3_^−^-activated PmrA dimer do not establish stable contacts in the presence of DNA. Therefore, the formation of an REC–DBD interface may be only transient and not stable enough to show two sets of NMR resonances. As expected, NMR CPMG relaxation dispersion experiments reveal that several interface methyl groups exhibit similar slow timescale motions in the presence of DNA. The transient formation of the interface may reduce the flexibility of the whole complex for successful crystallization. Normally, the transient conformers are difficult to study by conventional biophysical techniques and NMR is particularly useful in providing structural information on the invisible states. However, in the case of the PmrA–DNA complex, the transient conformer with a stable REC–DBD interface in the upstream protomer may be more suitable for crystallization than most of the other conformers with random REC–DBD interactions.

**Figure 8:**
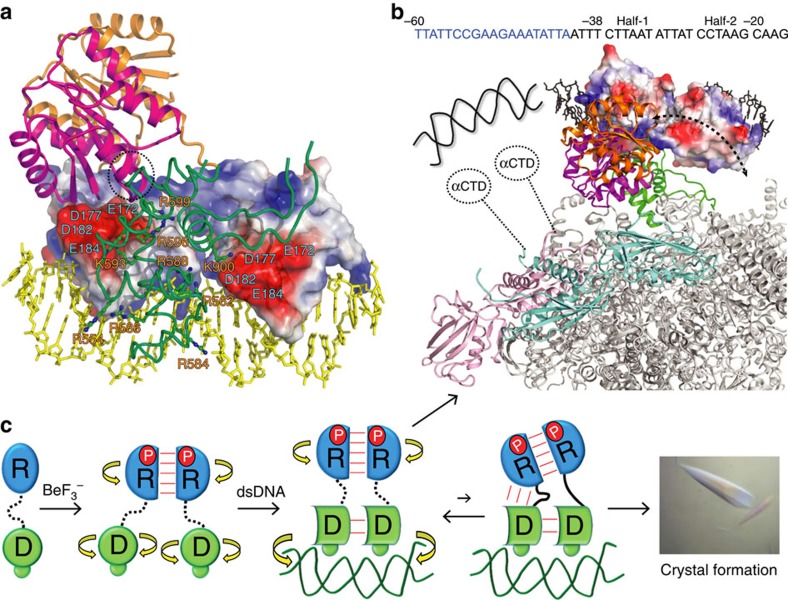
REC–DBD interdomain dynamics of PmrA and its possible role in RNAPH interaction. (**a**) The docking model of PmrA–DNA–RNAPH complex with the REC of PmrA-1 is in magenta, PmrA-2 in orange and DNA in yellow. Surface charge distributions of two DBDs show the acidic patches. The basic patches on σ_4_ and β-flap tip helix of RNAPH (green) are shown in sticks. (**b**) Top view of the model with two α-subunits of RNAPH in pink and cyan and others in grey, showing that with stable REC–DBD interface, only the REC on PmrA-1 can interact with the RNAPH. However, if the REC dimer is connected to DBD with flexible linkers, the dimer can tumble freely and find the best orientation to interact extensively with RNAPH. In addition to these interactions, the C-terminal domain of the α-subunit of RNAPH may interact with the upstream DNA or with the PmrA–DNA complex. (**c**) A cartoon diagram summarizes the structure and dynamics of PmrA in transcription regulation. The REC is in blue and DBD in green. The phosphoryl analogue BeF_3_^−^ is denoted as P. Linkers with high flexibility are shown as dotted lines and low mobility as solid lines. The interactions between domains are shown as red lines and the domains that can rotate independently by yellow curved arrows.

To better understand the roles of the REC–DBD interface in transcription activation, we dock the *E. coli* RNAPH^18^ to the PmrA–DNA complex structure on the basis of the crystal structure of the σ_4_-β-flap tip helix chimer/PhoB–DBD/DNA ternary complex^29^ (Supplementary Fig. 14). In the PhoB–DBD ternary complex structure, two PhoB–DBDs recognized the two half sites in the head-to-tail orientation and the σ_4_-β-flap tip helix chimer contacted the −35 element as well as the PhoB–DBD at the half-1 site. In the PmrA–DNA complex structure, two PmrA DBDs bind to promoter DNA sequences in a head-to-tail manner and the −35 element also locates at the half-1 site of the *pbgP* promoter^30^. With the similarity in promoter recognition between PmrA and PhoB, the PhoB–DBD ternary complex is a suitable bridge for the docking of RNAPH, although we have no direct evidence that PmrA interacts with σ_4_ RNAPH as does PhoB. In the docking model of the PmrA–DNA–RNAPH complex, the σ_4_ from RNAPH fits complementarily to the interface formed by the two PmrA DBDs. The acidic patches (Glu^172^, Asp^177^, Asp^182^ and Glu^184^) on the transactivation loop of two DBDs face the patch of basic residues from the σ_4_ and the β-flap tip helix (Fig. 8a). However, in another view of the docking model (Fig. 8b), with extensive REC–DBD interactions in PmrA-1, only the REC of PmrA-1 contacts with the RNAPH. Instead, with flexible linkers, the REC dimer can search for the best orientation to interact with the RNAPH with a larger interface when the DBDs are bound with the promoter DNA (Supplementary Movie 1). The REC–DBD interface seems not to play an important role in the contact between BeF_3_^−^-activated PmrA and the RNAPH, which agrees with the β-galactosidase reporter assay findings of PmrA variants with altered interface residues and suggests that the formation of a stable REC–DBD interface is not crucial for activating downstream gene transcription. This model suggests a direction for future investigation in that the REC–DBD interdomain dynamics and the DBD–DBD interface of PmrA may help in the formation of the initial closed promoter complex for transcription initiation.

In conclusion, we summarize the structure and dynamics of PmrA in transcription regulation (Fig. 8c). From NMR studies in solution, the linkers of BeF_3_^−^-activated PmrA are flexible and the RECs and DBDs tumble separately with or without DNA. CPMG relaxation dispersion NMR detects a transiently populated REC–DBD interaction (with 11% population and *k*_ex_∼560±49 s^−1^) in the presence of DNA. This transient interaction may reduce the dynamics of the PmrA–DNA complex and facilitate the formation of crystals, for a successful determination of the asymmetric PmrA dimer structure in complex with DNA. In this complex structure, the DNA basically exhibits a B-form–like conformation with a curvature of ∼40°; two BeF_3_^−^-activated RECs form a symmetric head-to-head dimer mediated by the α4–β5–α5 interface, and two DBDs, with Asn^188^, Asn^196^ and Arg^210^ recognizing DNA bases specifically, bind to the half-1 and half-2 sites to constitute a head-to-tail interface. The asymmetric arrangement of RECs and DBDs establishes extensive contacts between the REC and DBD in the upstream protomer with a stabilized turn-like conformation in the linker. However, β-galactosidase reporter assay of PmrA variants with altered interface residues suggests that the formation of the REC–DBD interface is not crucial for activating downstream gene transcription. The structure model of the PmrA–DNA–RNAPH complex suggests that when the basic residues from the RNAPH σ_4_ domain fit complementarily to the negatively charged surface of two DBDs, greater REC–DBD interdomain dynamics allow the REC dimer to rotate more freely to adopt a suitable orientation that can best contact with the RNAPH to form the initial closed promoter complex for transcription initiation.

In addition to the RNAPH σ_4_ domain, the interactions between the C-terminal domain of the α-subunit of RNAPH and OmpR or some other transcription activators were described^31^,^32^,^33^,^34^,^35^. To elucidate the initiation steps for transcription activation by PmrA, the interactions between PmrA–DNA and the σ_4_ or C-terminal domain of the α-subunit of RNAPH remain to be characterized, which is in progress.

## Methods

### Preparation of recombinant proteins and oligonucleotides

The DNA fragment encoding WT-PmrA from *K. pneumoniae* was cloned into a pET-29b(+) (Novagen) vector and transferred in *E. coli* strain BL21(DE3) with an extra Met residue at the N terminus and an additional LEHHHHHH tag at the C terminus for purification. The PmrA and WT-PmrA variants were generated by the QuickChange site-directed mutagenesis protocol (Stratagene) and confirmed by DNA sequencing. To prepare the methyl-protonated {Ile(δ1 ^13^CH_3_), Leu(^13^CH_3_, ^12^CD_3_), Val(^13^CH_3_, ^12^CD_3_)} U-[^2^H, ^13^C, ^15^N] sample, cells were grown in D_2_O containing M9 minimal medium supplemented with ^15^NH_4_Cl (1 g l^−1^), ^13^C, ^2^H-glucose (3 g l^−1^) at 37 °C. Precursors (α-keto-3-methyl-d_3_-butyric acid-4-^13^C and 2-ketobutyric acid-4-^13^C) were added to cells once *A*_600_ reached 0.8. Recombinant protein was purified by use of nickel-nitrilotriacetic acid affinity resin^19^ (Qiagen, Hilden, Germany). The purity of samples was checked with use of Coomassie-blue–stained SDS–polyacrylamide gel and was >95%. The protein samples were activated by adding 5.3 mM BeCl_2_, 35 mM NaF and 7 mM MgCl_2_ (ref. 19). Double-stranded DNA was prepared by mixing an equal amount of two complementary oligonucleotides in 20 mM sodium phosphate and 30 mM NaCl at pH 6.0, heating to 95 °C for 30 min and cooling slowly to room temperature, then further purified on a Mono-Q 5/50 GL column (Amersham Biosciences) with elution by NaCl concentration gradient from 0.1 to 1 M. The final concentration of DNA was calculated by ultraviolet absorbance at 260 nm.

### Crystallization and data collection

For crystallization screens, the BeF_3_^−^-activated PmrA dimer was mixed with a series of various-length DNAs at a 1:1 molar ratio in buffer containing 20 mM Tris-HCl, pH 8.0, 100 mM NaCl and 2.65 mM BeF_3_^−^. Initial crystallization trials involved use of commercial kits (Hampton Research, Jena Bioscience, Molecular Dimensions, Emerald Bio and Qiagen) with the sitting-drop vapor-diffusion method^36^. Small crystals of PmrA–25 bp and -26 bp complex appeared after 3 days in the commercial kit condition. After modification, hexagonal pyramid-shaped crystals of the PmrA–26-bp DNA complex grew under the condition of 0.1 M 3-(cyclohexylamino)-1-propanesulfonic acid, pH 9.8, 0.8 M sodium acetate and 12% (w/v) polyethylene glycol 1,000. The crystal diffracted to 9 Å at the beginning, so we used post-crystallization treatment to improve diffraction quality^37^. The coverslips were transferred to a reservoir containing 4 M NaCl as a dehydrating solution for 50 min. Dehydration improved the crystal diffraction remarkably to 3.8 Å in resolution. Native diffraction data were collected at wavelengths of 0.9000 Å by use of a Bruker-AXS DIP-6040 CCD detector on beamline BL44XU at SPring-8. To solve the phase problem, we produced crystals of selenomethionine-derivative PmrA in complex with 26-bp DNA and collected data. A multi-wavelength anomalous diffraction data set was collected from a single Se-labelled PmrA–DNA complex crystal at wavelengths 0.97923 Å (Se-edge), 0.96400 Å (Se-remote) and 0.97905 Å (Se-peak) by use of the Rayonix MX300HE CCD area detector at the National Synchrotron Radiation Research Center, Taiwan (beamline BL15A1). X-ray diffraction data were integrated and scaled by use of the HKL2000 package^38^. The crystal belongs to the hexagonal P3_1_21, with cell dimensions of *a*=*b*=162.1 Å and *c*=131.9 Å.

For the PmrA–25-bp DNA complex, plate-shaped crystals appeared under the condition of 0.1 M Tris-HCl, pH 7.5, 0.8 M ammonium acetate and 20% (w/v) polyethylene glycol 3,350. Crystal dehydration was also used to improve the resolution to 3.2 Å. Native crystal data were collected from a single PmrA–DNA complex crystal at wavelengths of 1.0000 Å by use of the ADSC Quantum-315 CCD area detector at the National Synchrotron Radiation Research Center, Taiwan (beamline BL15A1). X-ray diffraction data were integrated and scaled by use of the same programme. The crystal belongs to the orthorhombic C222, with cell dimensions of *a*=194.3 Å, *b*=250.8 Å, and *c*=108.9 Å. All of the diffraction data are in Table 1.

### Structural determination and refinement

The experimental phase of the PmrA–26-bp DNA complex was determined by the multi-wavelength anomalous diffraction method with the programme AutoSol from the PHENIX suite. The programs COOT^39^ and PHENIX^40^ were used for model building and refinement, respectively. The final model for the PmrA–26-bp DNA complex contained two PmrA molecules (219 residues in chain A and B), one double-stranded DNA molecule (26 nucleotides in chain C and D), two BeF_3_^−^ and two Mg^2+^ atoms. The structure was refined to a final *R*_work_ and *R*_free_ of 17.8% and 22.9%, respectively. The Ramachandran plot outlier of the PmrA–26-bp DNA is located on the α8–β9 loop (Ile^201^ of chain B). The structure of the PmrA–25-bp DNA complex was solved by using molecular replacement as implemented in PHENIX^40^ with the PmrA REC domain, DBD domain and 25-bp DNA from the PmrA–26-bp complex structure separately as search models. Model rebuilding and refinement involved the programme described above. The final model contained four PmrA molecules (219 residues in chain A, B, E and F), two double-stranded DNA molecules (25 nucleotides in chain C, D, G and H), four BeF_3_^−^, four Mg^2+^ atoms, and 86 water molecules in the asymmetric unit. The structure was refined to a final *R*_work_ and *R*_free_ of 18.1% and 23.2%, respectively, with good stereochemistry. The Ramachandran plot showed that 97.1% of residues lay within the most favoured regions, with no residues in the generously disallowed region. Detailed refinement parameters are in Table 1.

### NMR resonance assignment

All protein samples (∼0.6 mM) were prepared in buffer containing 20 mM Tris-HCl, 100 mM NaCl and 5 mM BeF_3_^−^ at pH 8.0. The NMR spectra were acquired at 310 K on Bruker AVANCE 600 and 850 MHz spectrometers equipped with a z-gradient TXI cryoprobe (Bruker, Karlsruhe, Germany). Backbone resonance assignment of the BeF_3_^−^-activated PmrA was based on the assignments of stand-alone REC and DBD combined with TROSY-NHCACB, TROSY-HNCO and TROSY-HN(CA)CO spectra. Assignment of BeF_3_^−^-activated PmrA in complex with 25-bp DNA involved TROSY-HNCA and HNN-NOESY^22^. The ‘out-and-back' 3D HMCM(CG)CBCA experiment^21^ was acquired on a 0.4-mM methyl-protonated {Ile(δ1 ^13^CH_3_), Leu(^13^CH_3_, ^12^CD_3_), Val(^13^CH_3_, ^12^CD_3_)} U-[^2^H, ^13^C, ^15^N] sample of PmrA for assigning methyl groups. Stereospecific assignment of methyl groups of Leu and Val residues was as described^41^. Briefly, we used the mixture of 10% ^13^C-glucose and 90% unlabelled glucose as the carbon source for the expression of the PmrA sample and analysis of the distribution of ^13^C labels of the methyl groups in Leu and Val residues on a 2D ^1^H, ^13^C HSQC spectrum. The weighted CSPs for backbone ^15^N and ^1^H_N_ resonances were calculated by the equation Δ*δ*=[((Δ*δ*_HN_)^2^+(Δ*δ*_N_/5)^2)^/2]^0.5^ and for methyl ^13^C and ^1^H resonances by the equation Δ*δ*=[((Δ*δ*_H_)^2^+(Δ*δ*_C_ × 0.3)^2)^/2]^0.5^. For Leu and Val residues, the methyl CSP was the mean value of two methyl groups. All NMR spectra were processed by use of NMRPipe^42^ and analysed by use of NMRView^43^.

### *R*_1_ and *R*_2_ dynamics and CPMG relaxation dispersion experiments

The longitudinal (*R*_1_) and transverse (*R*_2_) relaxation rates were measured in duplicate at 310 K on a Bruker 850 MHz machine. The inversion recovery delays in the *R*_1_ experiments were 10, 20, 40, 70, 120, 200, 300, 500, 700, 1,000 and 1,500 ms. The *R*_2_ relaxation experiment were obtained with durations of 17.2, 34.3, 51.5, 68.6, 85.8, 102.9, 120.1 and 137.2 ms. The repetition delay for all *R*_1_ and *R*_2_ experiments were set to 2 s. CPMG relaxation dispersion experiments were acquired on [Ile(δ1 ^13^CH_3_), U-(^2^H, ^15^N)] PmrA–25-bp DNA complex or [Leu(^13^CH_3_, ^12^CD_3_), Val(^13^CH_3_, ^12^CD_3_), U-(^2^H, ^15^N)] PmrA–25-bp DNA complex at 310 K with 600 and 850 MHz NMR machines. The methyl-TROSY ^13^C–^1^H multiple quantum relaxation dispersion experiments were acquired with *ν*_CPMG_ of 50, 100, 150, 200, 250, 300, 400, 500, 700 and 1,000 Hz and a total time of 20 ms (*T*_relax_)^27^. The spectra with *ν*_CPMG_ of 50, 100 and 700 Hz were collected twice to estimate experimental errors. A reference spectrum was also acquired by omitting the CPMG intervals.

The relaxation parameters were analysed by extracting peak heights by an automated routine in NMRView^43^. For relaxation dispersion, the effective decay rate (*R*_2,eff_) was calculated as *R*_2,eff_=(−1/*T*) ln[*I*(*ν*_CPMG_)/*I*(0)], where *I*(*ν*_CPMG_) and *I*(0) are the intensities of peaks recorded with and without the CPMG intervals, respectively. CPMG relaxation dispersion measurements at both 600 and 850 MHz were fitted individually by the software relax^28^ to a two-site exchange process. The detailed auto-analysis process can be found in the online manual for relax (http://www.nmr-relax.com/docs.html). Two models, MMQ CR72 and NS MMQ 2-site, were fitted and Akaike's model selection (AIC)^44^ was performed to judge statistical significance of the models. Successful fitting of relaxation dispersion profiles yielded the populations (p_A_ and p_B_), exchange rate constants (*k*_ex_=*k*_AB_+*k*_BA_) and the ^1^H and ^13^C chemical shift differences between the two states. Because of limited concentration of the PmrA–DNA complex, these parameters were reported only when *R*_ex_, defined as *R*_2,eff_(50 Hz)−*R*_2,eff_(1,000 Hz), exceeded 3 s^−1^ (Supplementary Table 4).

### Fluorescence polarization measurements

The oligonucleotides (25 bp) for fluorescence polarization experiments were labelled with 6-carboxyfluorescein at the 5′ position. The indicated amount of proteins was added to the well containing 6 nM of 6-carboxyfluorescein-labelled DNA in 10 mM sodium phosphate and 15 mM NaCl at pH 7.0 and 298 K. Reactions were measured three times by use of a SpectraMax Paradigm plate reader (Molecular Devices, CA, USA) with excitation wavelength 485 nm and emission wavelength 535 nm. The binding curves were fitted by a one-site binding model. Data were analysed and plotted by use of GraphPad Prism 6 (San Diego, CA, USA).

### β-Galactosidase reporter assay

The DNA fragment (∼500 bp) containing the upstream region of *K. pneumoniae pbgP* gene was PCR-amplified and inserted in front of a promoter-less *lacZ* gene in the plasmid placZ15 (ref. 45). The resulting reporter plasmid was mobilized to *K. pneumoniae* CG43S3-Δ*lacZ* strain by conjugation. Then the plasmids that express PmrA, WT-PmrA or its variants were transformed into the *K. pneumoniae* cells that carry the reporter plasmid. Individual transformants were cultured at 37 °C in LB medium containing isopropyl-β-D-thiogalactoside (1 mM ml^−1^), kanamycine (30 μg ml^−1^) and chloramphenicol (34 μg ml^−1^) to mid-logarithmic phase and harvested by centrifugation. β-galactosidase activity was measured and expressed as Miller units^46^ (1,000 × Abs_420_/(*T* × *V* × *A*_600_)), where *T* and *V* indicate reaction time in minutes and volume of culture in millilitres, respectively. Graphs represent the average values and standard errors from triplicate measurements.

### Docking PmrA–DNA complex structure with the *E. coli* RNAPH

The docking involved use of PyMOL^47^. The starting structures for docking were the PmrA–DNA complex structure (complex-1) and the *E. coli* RNAPH structure^18^ (PDB-ID: 4IGC). The crystal structure of the σ_4_-β-flap tip helix chimer/PhoB–DBD/DNA ternary complex^29^ (PDB: 3T72) was used as a model to drive the structure-based alignment. We selected the residues for two PhoB–DBDs (residues 128 to 229) and two PmrA DBDs (residues 126 to 219) and executed the command align, which performs a sequence alignment followed by a structural superimposition and then carries out cycles of refinement to reject structural outliers found during the fit. The resulting r.m.s.d. value of the aligned C_α_ atoms was 2.35 Å. Then the residues (residues 532–609) of the RNAPH σ_4_ region and the σ_4_ in the PhoB–DBD ternary complex were selected and aligned (r.m.s.d.=2.32 Å) to create a possible interacting model of the PmrA–DNA–RNAPH complex based on the crystal structure of the σ_4_-β-flap tip helix chimer/PhoB–DBD/DNA ternary complex. The process to generate the docking model is depicted in Supplementary Fig. 14.

## Additional information

**Accession codes:** Coordinates of PmrA–25 and –26-bp DNA complexes have been deposited in the RCSB Protein Data Bank with accession codes 4S04 and 4S05, respectively. The chemical shifts of BeF-activated PmrA with and without 25-bp DNA have been deposited in the Biological Magnetic Resonance Data Bank with accession codes BMRB-26535 and BMRB-26532, respectively.

**How to cite this article:** Lou, Y.-C. *et al.* Structure and dynamics of polymyxin-resistance-associated response regulator PmrA in complex with promoter DNA. *Nat. Commun.* 6:8838 doi: 10.1038/ncomms9838 (2015).

## Supplementary Material

Supplementary Information Supplementary Figures 1-14 and Supplementary Tables 1-4

Supplementary Movie 1 The REC-DBD interdomain dynamics of PmrA. When the PmrA-DNA complex (PmrA-1, PmrA-2 and DNA are in green, yellow and blue, respectively) is recognized by the σ4 domain of RNAPH, greater REC-DBD interdomain dynamics allow the REC dimer to rotate more freely to adopt a suitable orientation that can best contact with the RNAPH to form the initial closed promoter complex for transcription initiation.

## Figures and Tables

**Table 1:** Data collection and refinement statistics for PmrA–DNA complexes.

**Data collection**	**PmrA–25-bp DNA**	**PmrA–26-bp DNA**	**SeMet PmrA–26-bp DNA**		
*Space group*	*C*222	*P*3_1_21	*P*3_1_21		
*Cell dimensions*					
*a, b, c* (Å)	194.33, 250.76, 108.94	162.64, 162.64, 131.71	162.12, 162.12, 131.85		
α, β, γ (°)	90, 90, 90	90, 90, 120	90, 90, 120		
			*Peak*	*Inflection*	*Remote*
Wavelength (Å)	1.00000	1.00000	0.97905	0.97923	0.96400
Resolution (Å)	30.0-3.2 (3.26-3.20)	30-3.8 (3.89-3.80)	50-4.4 (4.48-4.40)	50-4.4 (4.48-4.40)	50-4.4 (4.48-4.40)
*R*_merge_ (%)	8.9 (37.2)	6.6 (56.0)	11.3 (52.6)	11.2 (51.9)	10.7 (53.5)
*I*/σ*I*	16.4 (3.5)	16.3 (2.3)	20.3 (3.2)	20.4 (3.5)	20.0 (3.5)
Completeness (%)	99.3 (97.3)	99.5 (100.0)	97.1 (82.5)	97.0 (83.0)	96.8 (82.8)
Redundancy	4.6 (3.4)	3.2 (3.3)	6.1 (4.6)	6.0 (4.6)	6.0 (4.5)
					
*Refinement*					
Resolution (Å)	24.2–3.2	29.8–3.8			
No. of reflections	38,831	19,930			
*R*_work_/*R*_free_	18.1/23.2	17.8/22.9			
No. of atoms					
Protein	6,904	3,452			
DNA	2050	1066			
Ligand	20	10			
Water	86	—			
*B*-factors					
Protein	55.65	61.50			
DNA	68.38	99.64			
Ligand	51.59	49.35			
Water	33.94	—			
R.m.s.d.					
Bond lengths (Å)	0.012	0.037			
Bond angles (°)	1.830	1.443			

Data were collected on a single crystal for each data set. Values in parentheses are for highest resolution shell.
