# Supplementary material for: Structure and dynamics of polymyxin-resistance-associated response regulator PmrA in complex with promoter DNA
Source: Nat Commun. 2015 Nov 13;6:8838. doi: 10.1038/ncomms9838 (PMC4660055; doi:10.1038/ncomms9838)
Supplement: Supplementary Information — Supplementary Figures 1-14 and Supplementary Tables 1-4 [file ncomms9838-s1.pdf]

-

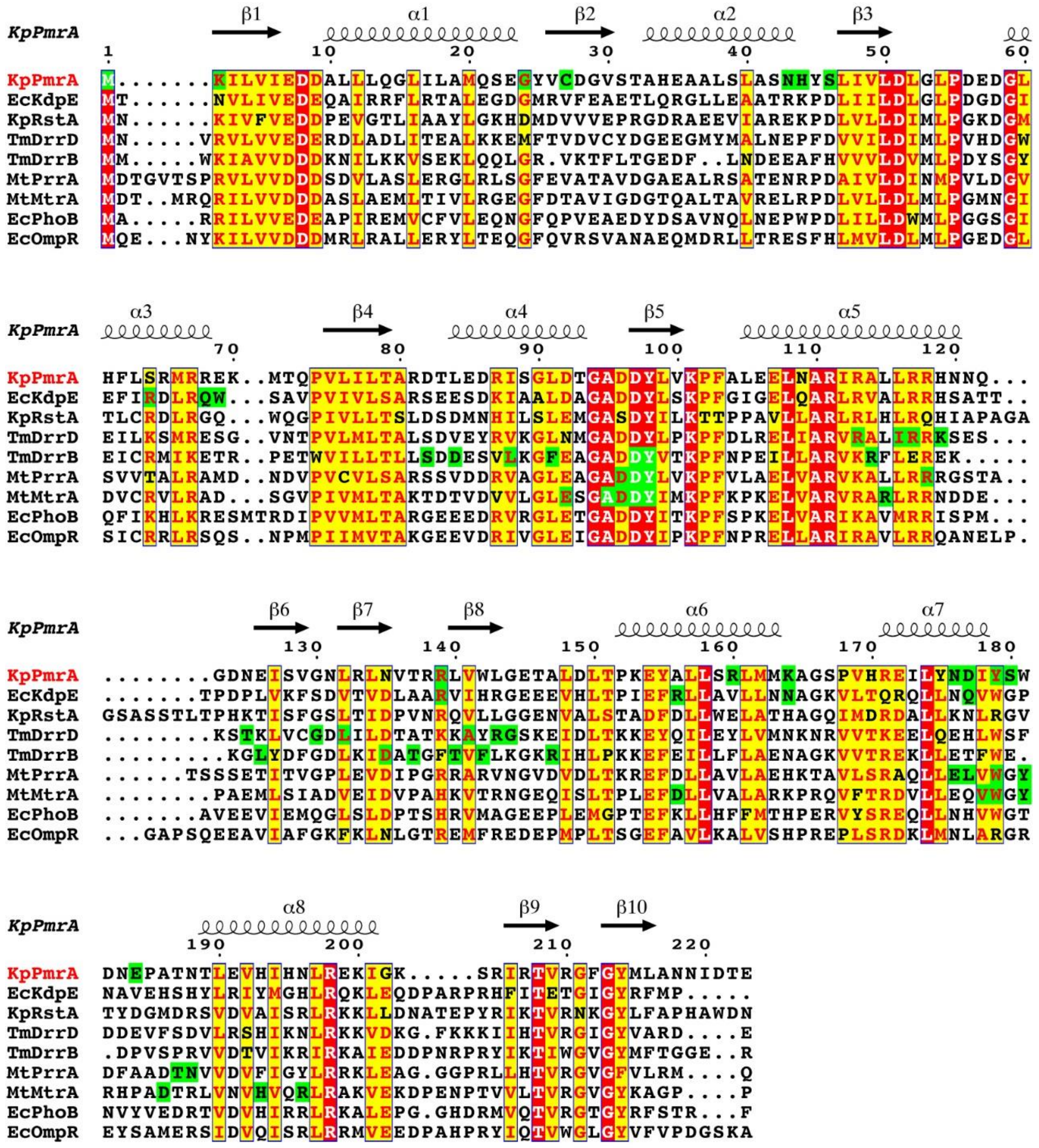

Supplementary Figure 1. Sequence alignment of OmpR/PhoB response regulators. They are *Klebsiella pneumoniae* PmrA (KpPmrA), *Escherichia coli* KdpE (EcKdpE), *K. pneumoniae* RstA (KpRstA), *Thermotoga maritime* DrrD (TmDrrD), *T. maritime* DrrB (TmDrrB), *Mycobacteria tuberculosis* PrrA (MtPrrA), *M. tuberculosis* MtrA (MtMtrA), *E. coli* PhoB (EcPhoB) and *E. coli* OmpR (EcOmpR). The secondary structures of KpPmrA are shown. The residues with green background color are involved in the REC-DBD interactions identified from crystal structures in inactive or active states.

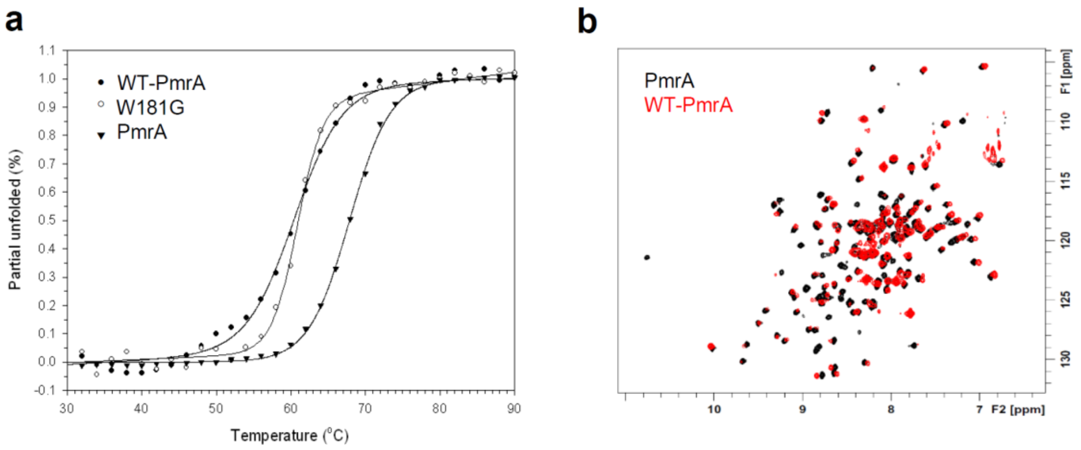

Supplementary Figure 2. Characteristics of WT-PmrA and PmrA. (**a**) Thermal denaturation curves of WT-PmrA (filled circles), W181G (open circles) and PmrA (filled triangles) acquired on a circular dichroism spectrometer (Aviv, Lakewood, N.J. USA) following changes of signals at 216 nm. PmrA exhibits higher thermal stability than the other two. (**b**) 2D ^{1}H, ^{15}N TROSY-HSQC spectra of WT-PmrA (red) and PmrA (black), show that both adopt a similar conformation.

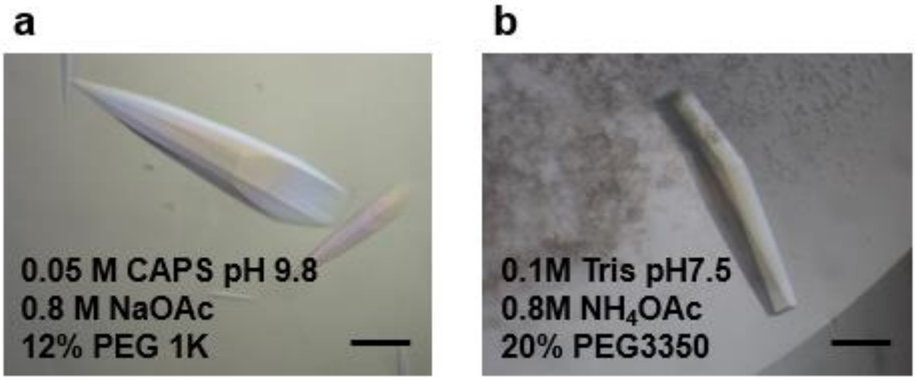

Supplementary Figure 3. Crystals of PmrA-DNA complexes. A series of various-length DNAs covering the *pbgP* promotor sequences were mixed with an equal amount of BeF_3^--activated PmrA for co-crystallization. Only the crystals of PmrA-26 bp DNA (**a**) and PmrA-25 bp DNA (**b**) are obtained. The conditions for crystal formation are indicated. Scale bars represent 100 μm.

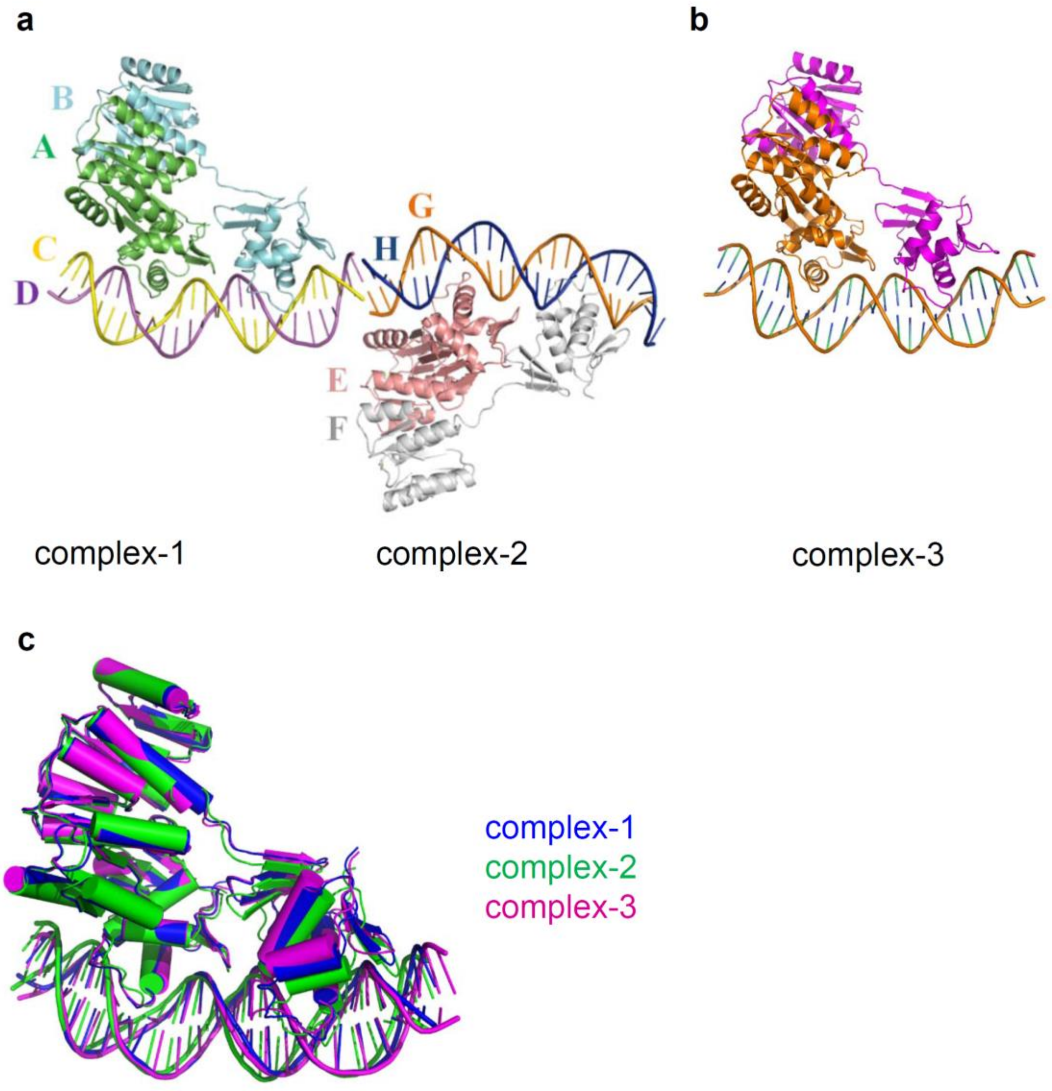

Supplementary Figure 4. Crystal structures of PmrA-DNA complexes. (**a**) The space group of the PmrA-25 bp DNA crystal is C222, with 2 copies of the protein-DNA complex (complex-1 and complex-2) in the asymmetric unit. The resolution of this complex structure is 3.2 Å. (**b**) The PmrA-26 bp DNA crystal has only one copy of the protein-DNA complex (complex-3) packed in a different space group $P3_121$ with a 3.8 Å resolution. (**c**) The 3 complex structures all contain a BeF_3^- -activated PmrA dimer (residues 1-219 modeled) binding to a double-stranded DNA (PmrA-1 binds to half-1 site and PmrA-2 half-2 site) with similar conformations. The root mean square deviations between C_α coordinates of the 3 complexes are listed in Supplementary Table 2.

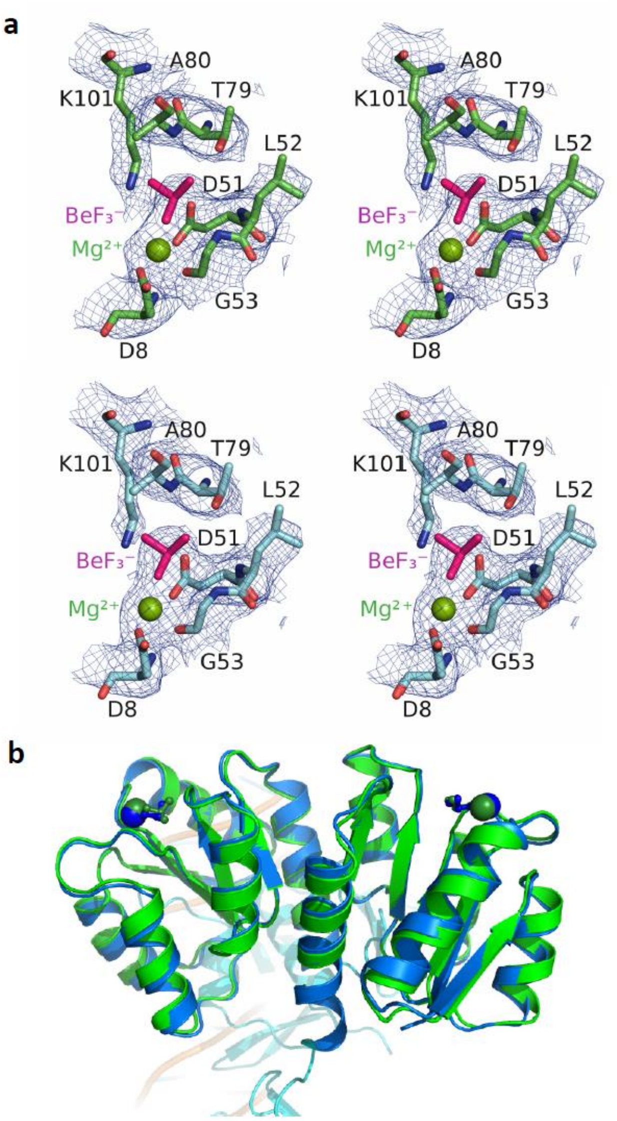

Supplementary Figure 5. Structures of the active sites and the REC dimer. (**a**) Stereo view of the active sites in PmrA-1 (green) and PmrA-2 (cyan). The electron density maps ($2F_o - F_c$) of the two protomers calculated at 1.0σ are shown with blue contours. Although the resolution is not high enough, the BeF_3^- and Mg^{2+} fit well to the contours, resulting in a similar occupancy in 2 active sites. The Mg^{2+} (green sphere) contacts Asp^{51}, Gly^{53}, Asp^{8} and BeF_3^-. The BeF_3^- moiety (magenta sticks) contacts Asp^{51}, Leu^{52}, Gly^{53}, Thr^{79}, Ala^{80} and Lys^{101}. (**b**) Two activated RECs form a symmetric dimer mediated by the α4-β5-α5 interface. Superimposition of the REC dimer in PmrA-DNA complex structure (blue) to the stand-alone REC dimer (blue). The RMDS value between two structures is 0.55 Å for C_α atoms.

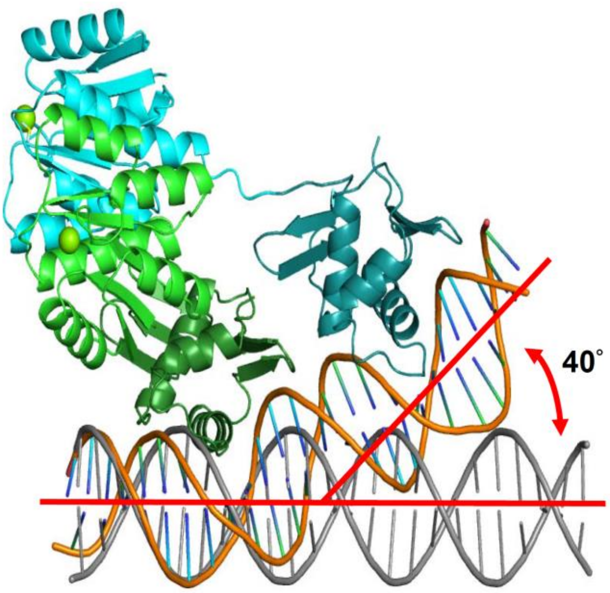

Supplementary Figure 6. Conformation of DNA. The DNA in the PmrA-25bp DNA complex structure bends around the protein with a curvature of approximately 40° as compared with the standard B-form DNA (gray).

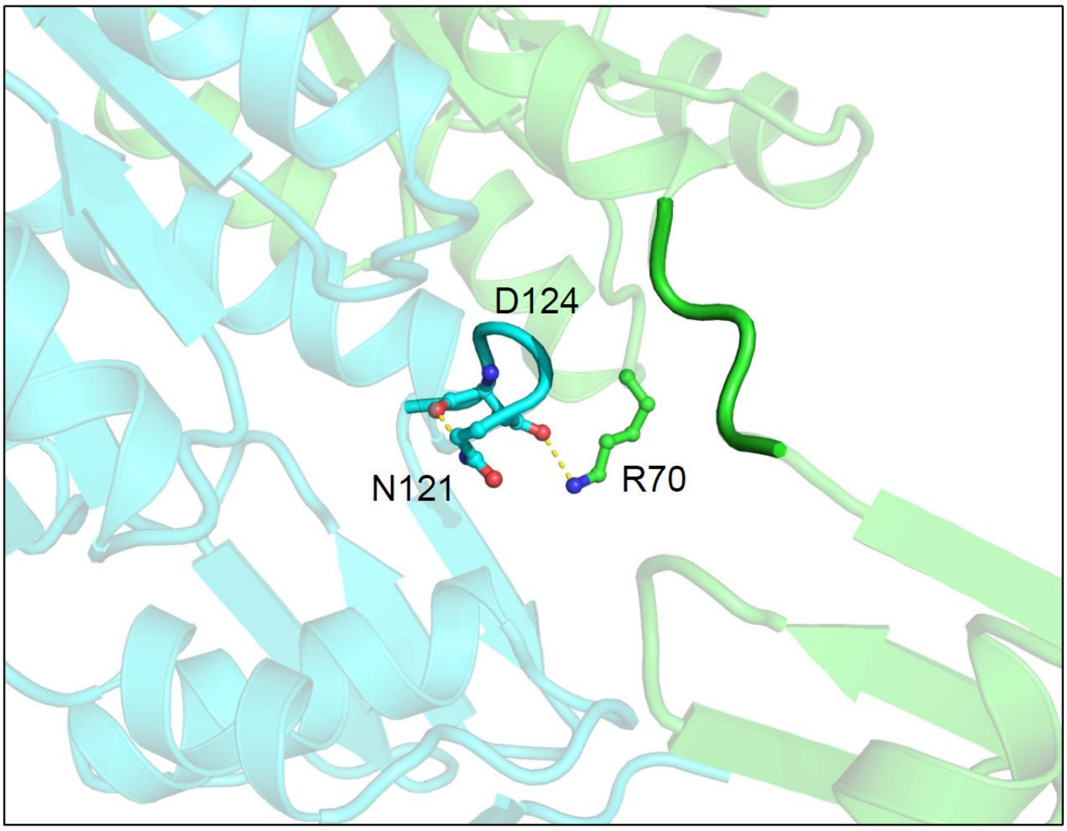

Supplementary Figure 7. Structure of the linkers. The linker in PmrA-1 (cyan) forms a turn-like conformation stabilized by an H-bond between Asp^{124} and Asn^{121} and a salt-bridge between Arg^{70} and Asp^{124}. In PmrA-2, the REC and DBD are linked by an extended linker (green) without any inter-domain interaction.

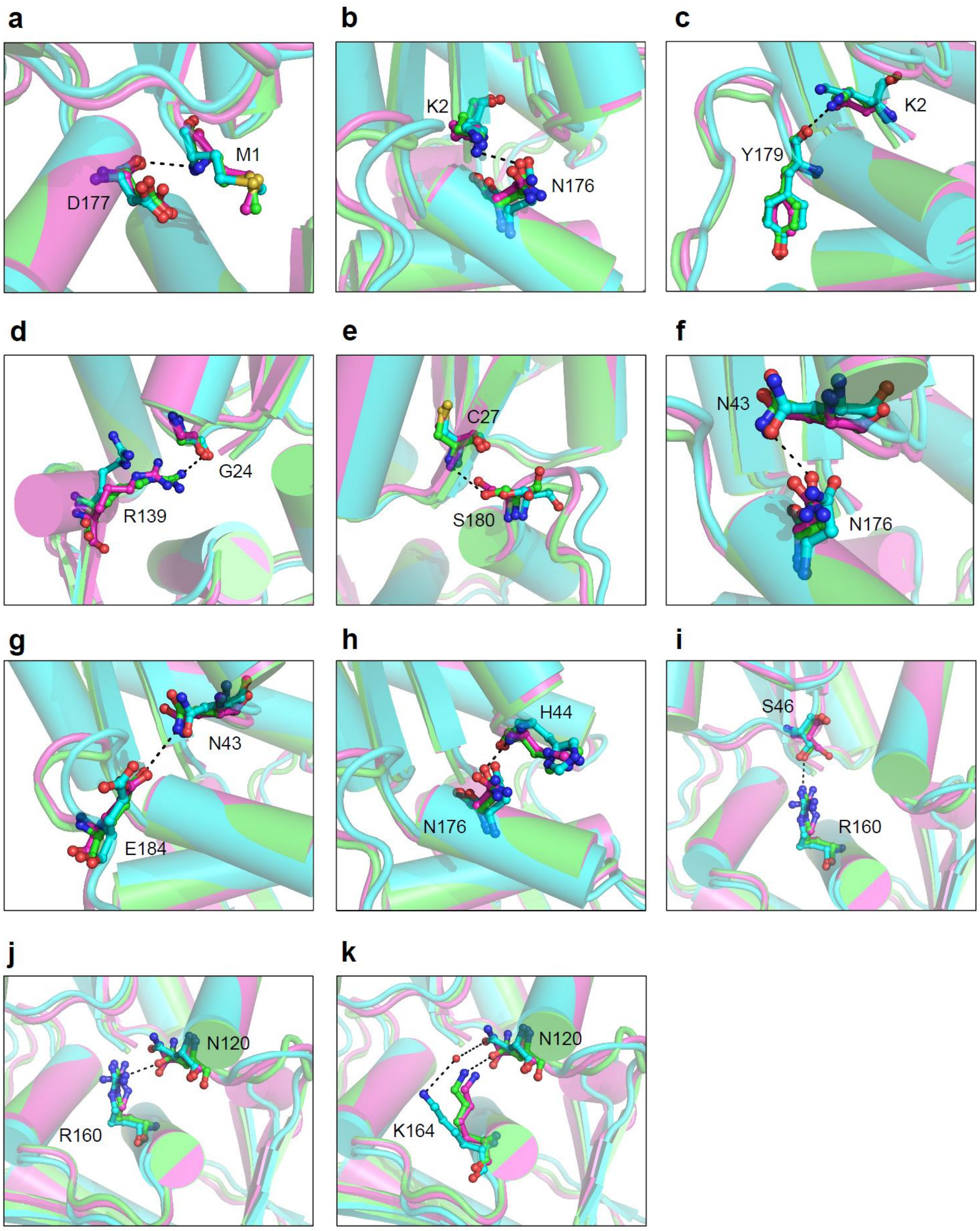

Supplementary Figure 8. The REC-DBD interactions in 3 PmrA-DNA complex structures. The interactions of 11 H-bonds in 3 complex structures are shown from (**a**) to (**k**). Complex-1, complex-2 and complex-3 are in green, cyan and magenta, respectively. Interacting residues are shown as sticks and balls with nitrogen, oxygen, and sulfur atoms in blue, red and yellow, respectively. The distances between H-bond donors and acceptors are listed in Supplementary Table 3.

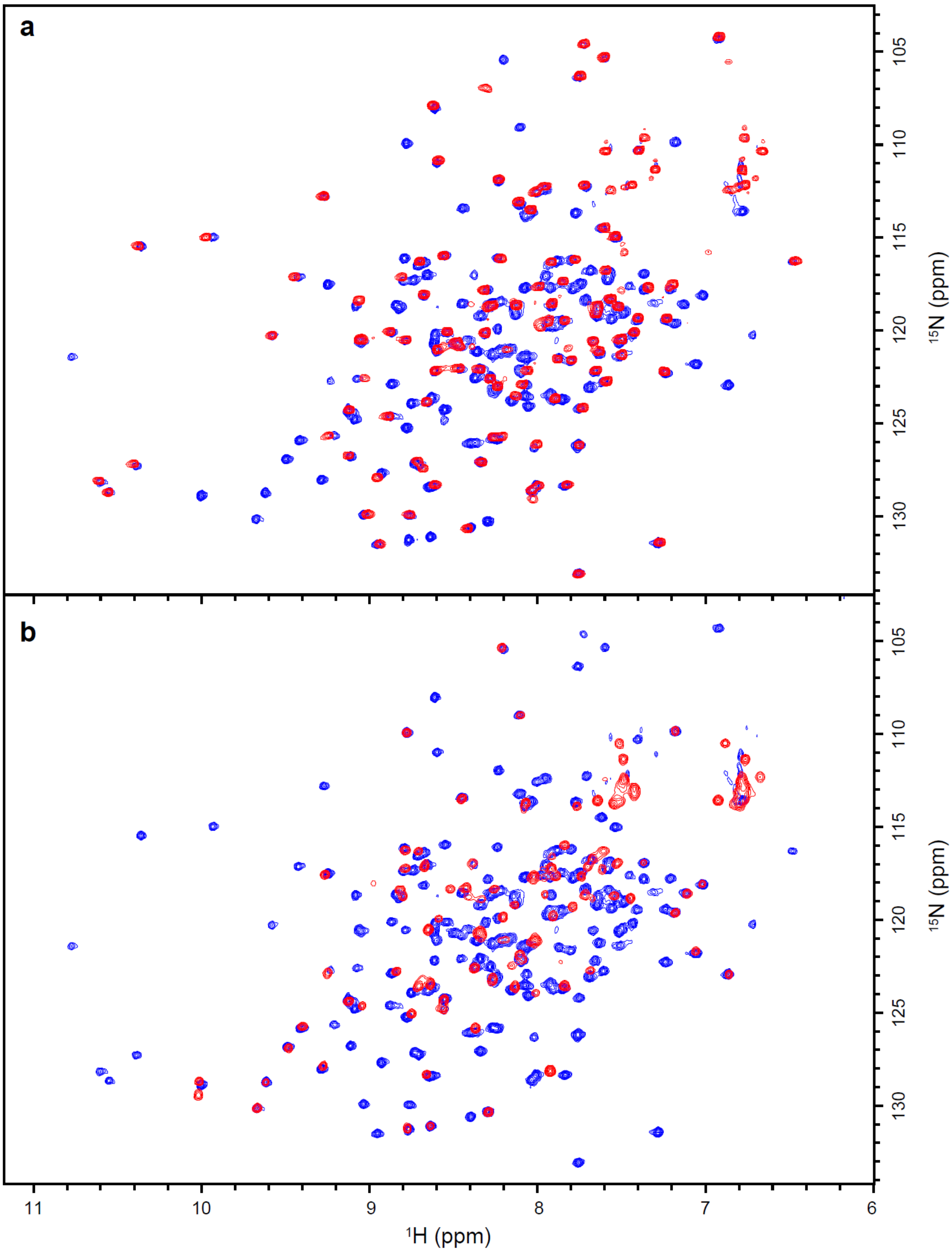

Supplementary Figure 9. Comparison between PmrA and stand-alone domains. The overlaid 2D ^{1}H, ^{15}N TROSY-HSQC spectra were acquired from BeF_3^--activated PmrA (blue), standalone BeF_3^--activated REC dimer (red in **a**) and standalone DBD domain (red in **b**). All samples were prepared at pH 8.0 and spectra were acquired at 310K.

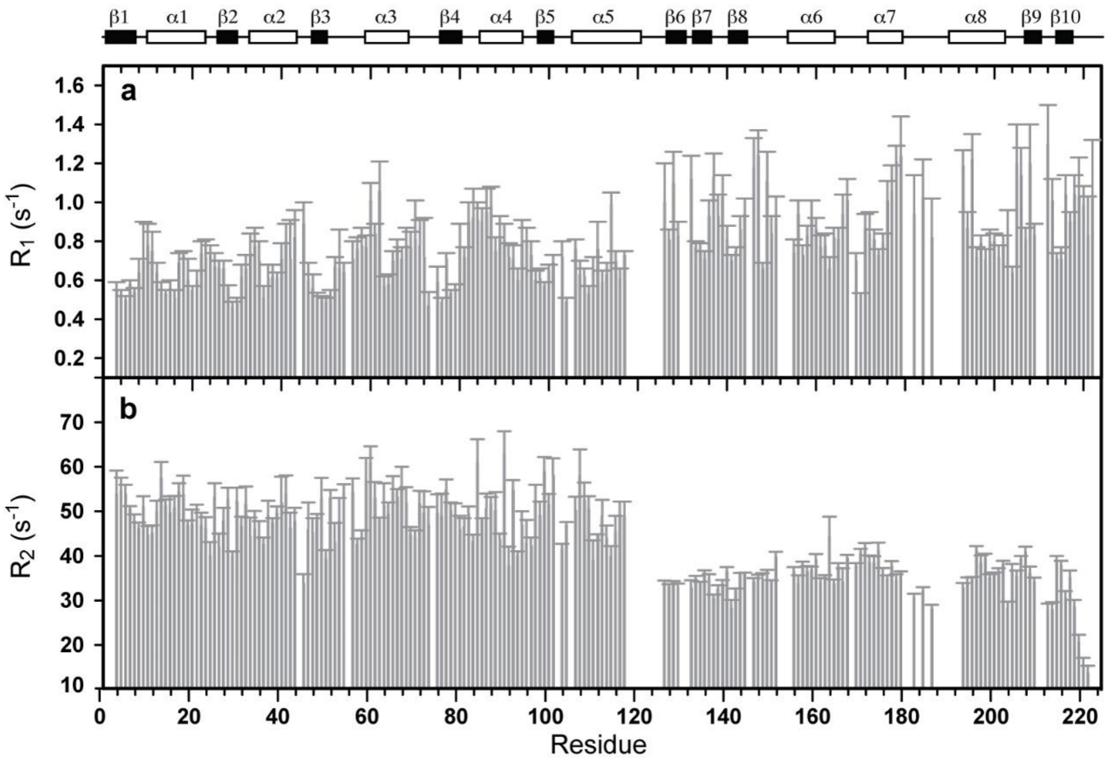

Supplementary Figure 10. Backbone dynamics of PmrA. The R_1 (**a**) and R_2 (**b**) relaxation rates of BeF_3^--activated PmrA. The average R_1 values are only slightly different for residues in REC and DBD domains, but the average R_2 rates are rather distinct, with values 48.9 s^{-1} and 34.2 s^{-1} for residues of REC and DBD, respectively. All experiments are recorded in duplicate. Error bars represent fitting errors.

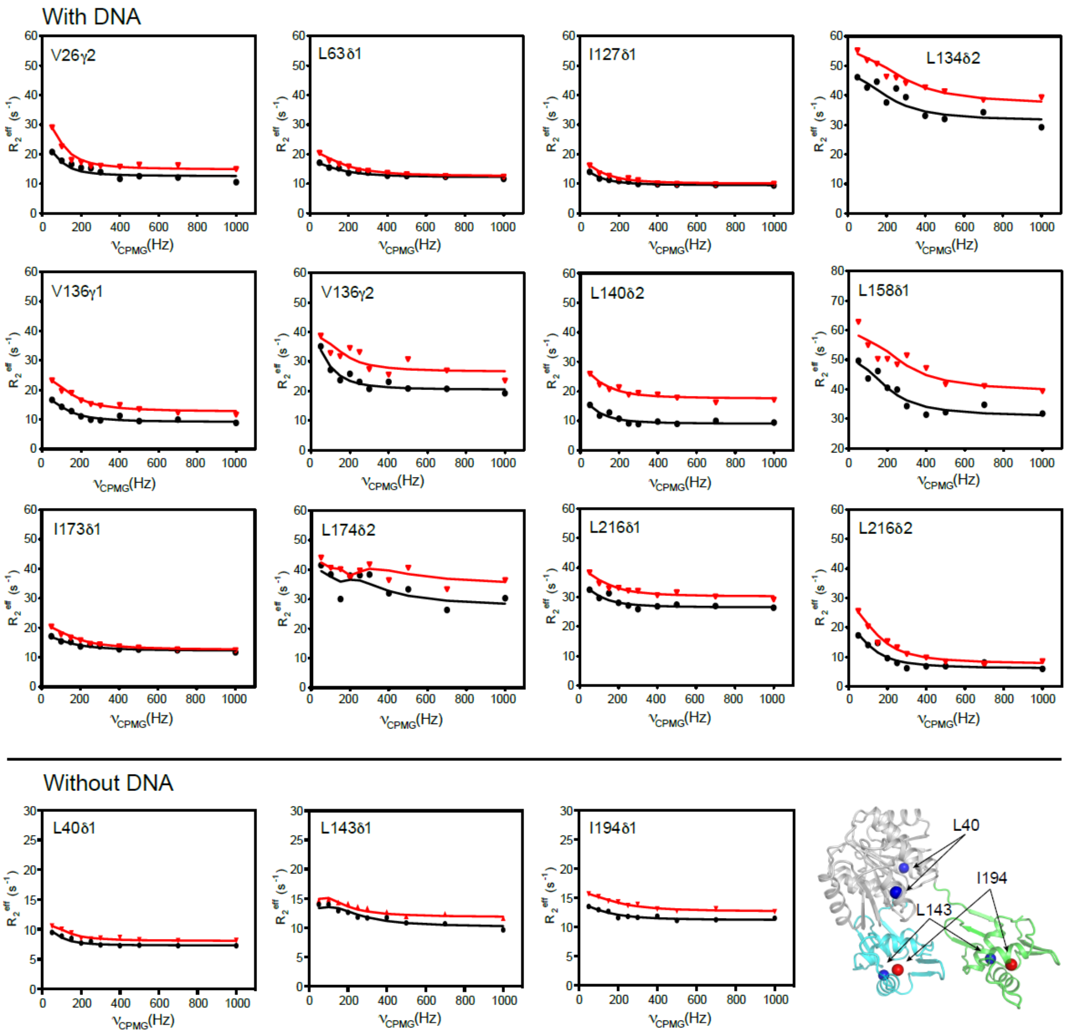

Supplementary Figure 11. CPMG relaxation dispersion NMR. All methyl-TROSY multiple-quantum relaxation dispersion profiles of BeF_3^--activated PmrA with and without DNA recorded at 600 (black) and 850 (red) MHz, 310K, with R_{ex}, defined as $R_{2,eff}$(50Hz) – $R_{2,eff}$(1000Hz), exceeds 3 s^{-1}. Lines show individual fits to a two-site exchange process. The methyl groups of BeF_3^--activated PmrA without DNA that exhibit slow dynamics are indicated by colored spheres, to show the amount of exchange rate constants (k_{ex}). The color codes are the same as Fig. 6b. All derived exchange parameters are in Supplementary Table 4.

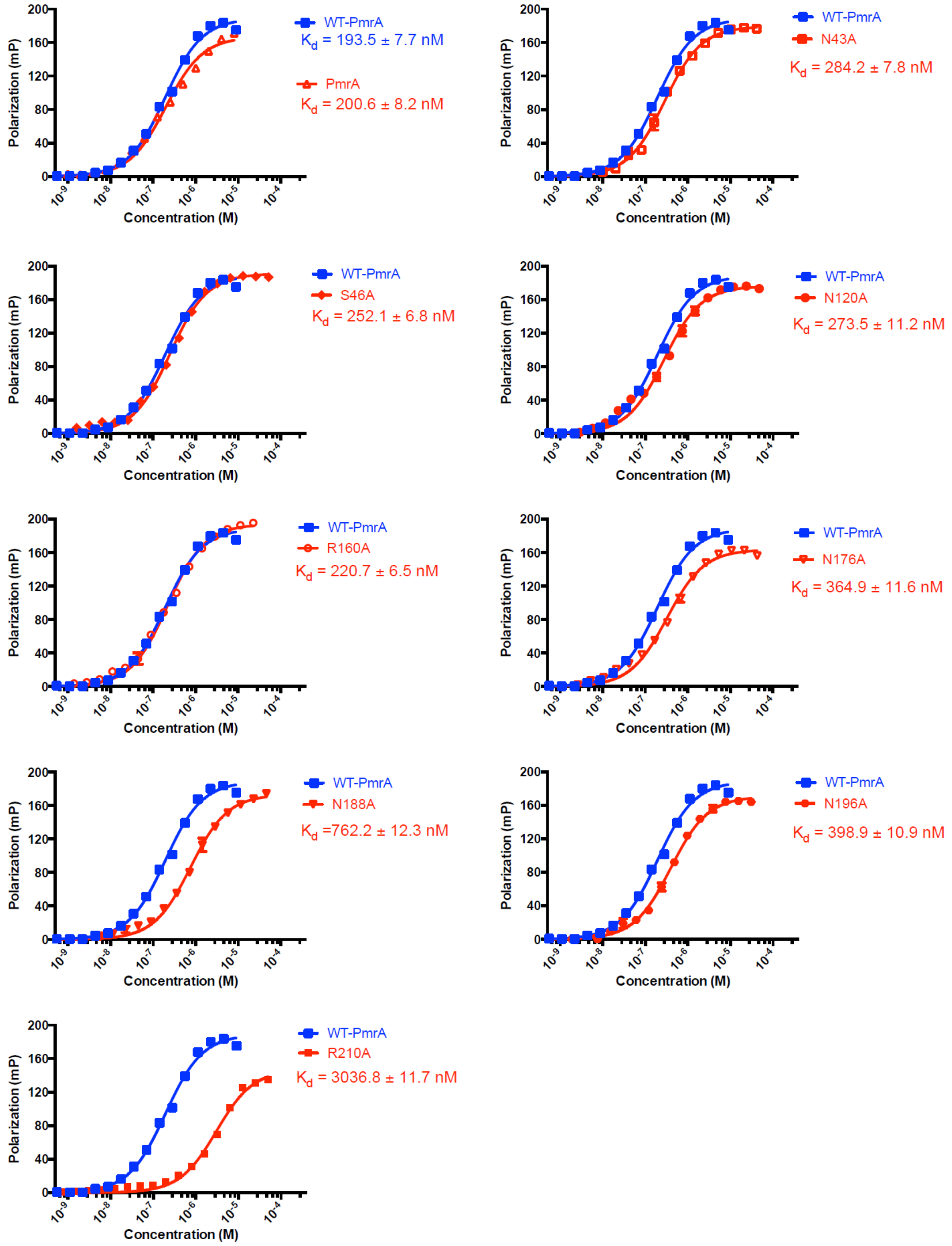

Supplementary Figure 12. Binding affinities obtained from fluorescence polarization experiments. The binding of PmrA, WT-PmrA or its variants to fluorescence-labeled DNA measured 3 times with excitation wavelength 485 nm and emission wavelength 535 nm in 10 mM sodium phosphate and 15 mM NaCl at pH 7.0, 298K. Error bars are defined as s.d. Binding affinities were derived from a one-site binding model by use of GraphPad Prism 6.

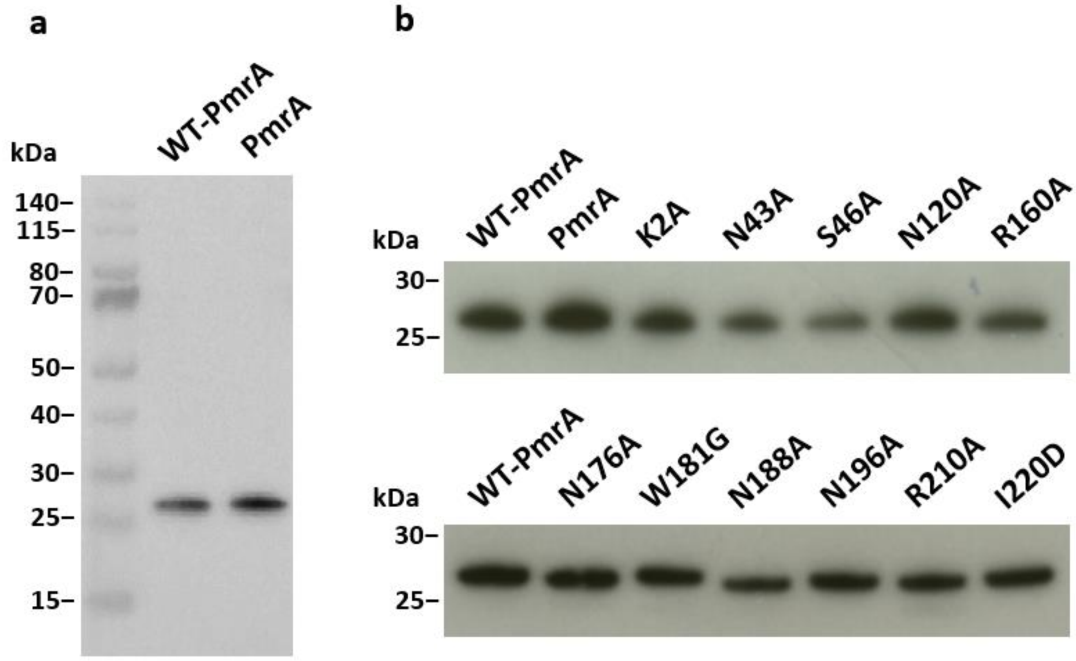

Supplementary Figure 13. Expression levels of all protein constructs. (**a**) Western blots of WT-PmrA and PmrA expressed in *K. pneumoniae* cells with Prestained Protein Ladder (Thermo) indicating the size of proteins. The image was acquired using a BioSpectrum 600 Imaging System (UVP). (**b**) After β-galactosidase reporter assay, *K. pneumoniae* cells (OD_{600} = 1.0) were sonicated, with supernatants subjected to SDS-PAGE, transferred to polyvinylidene difluoride membranes and probed with anti-His antibody (#MO-H40005.A Anogen; 1/5000), then horseradish-peroxidase conjugated (HRP) secondary antibodies (#31430 Thermo; 1/20000). Protein signals are detected by exposing the membranes to x-ray film after treatment with immobilon western chemiluminescent HRP substrate (Millipore). The mutant K2A, which precipitates greatly after activation with BeF_3^-, was not used in the fluorescence polarization experiment and the β-galactosidase reporter assay.

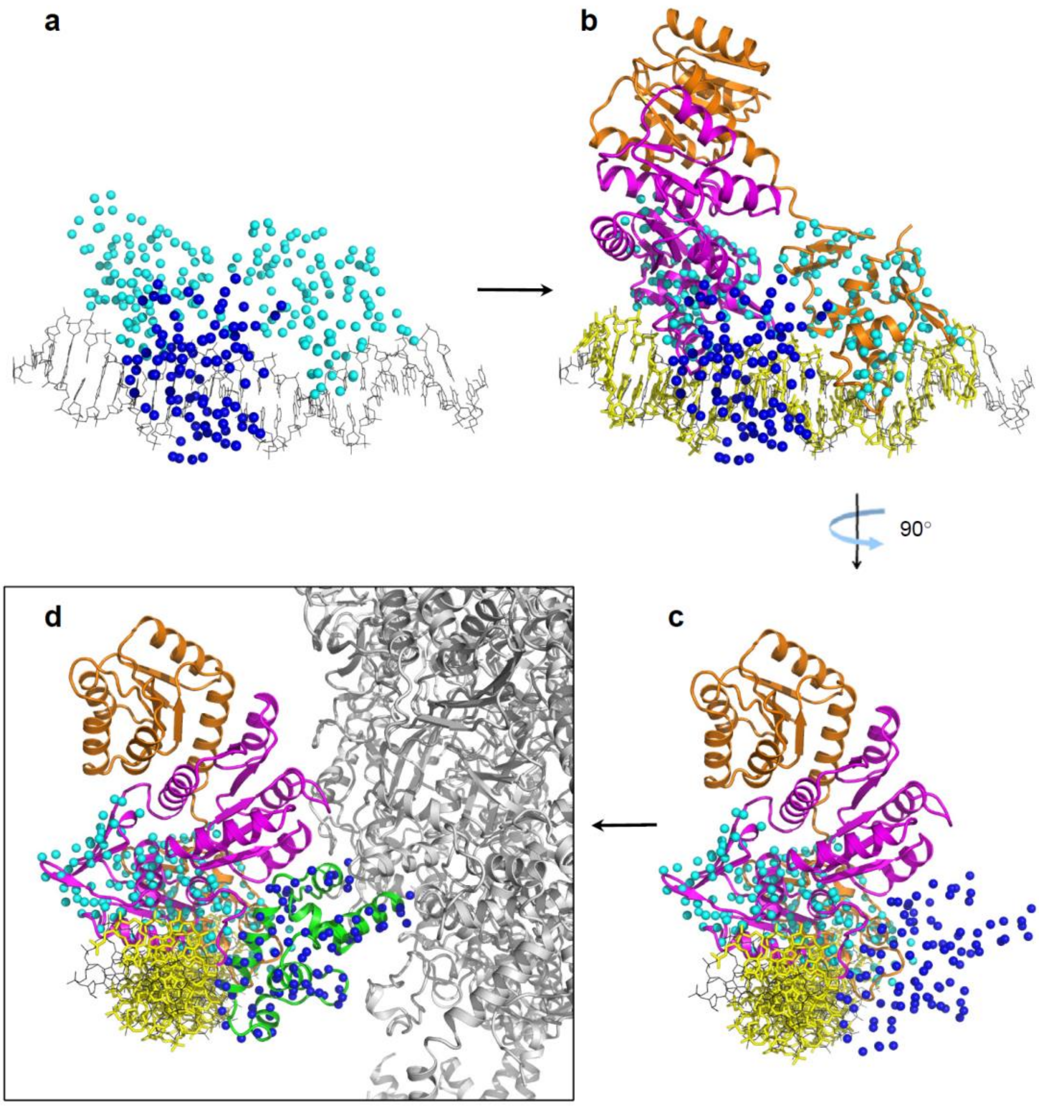

Supplementary Figure 14. The process to generate the docking model of PmrA-DNA-RNAPH complex. (**a**) The X-ray structure of σ_4-β-flap tip helix chimer/PhoB-DBD/DNA ternary complex (PDB: 3T72). DNA is shown as gray lines, The Cα atoms of 2 PhoB DBDs and σ_4-β-flap tip helix chimer are cyan and blue spheres, respectively. (**b**) We aligned the C_α atoms of 2 PhoB DBDs in this ternary complex to the DBDs of PmrA (RMSD = 2.35 Å). Two PmrA structures are in magenta and orange, respectively. DNA for PmrA is in yellow sticks. (**c**) Side-view of the docking model. (**d**) The C_α atoms of RNAPH σ_4 domain (PDB: 4IGC) were then superimposed to the σ_4 of PhoB-DBD ternary complex (RMSD = 2.32 Å) to generate the docking model of PmrA-DNA-RNAPH complex. The σ_4 domain of RNAPH is in green and others in gray.

Supplementary Table 1. The *pmra box* DNAs used for PmrA-DNA co-crystals.

DNA	Sequence
22bp	5′-TTCTTAATATTATCCTAAGCAG-3′<br>3′-CAAGAATTATAATAGGATTCGT-5′
24bp	5′-TTTCTTAATATTATCCTAAGCAAG-3′<br>3′-CAAAGAATTATAATAGGATTCGTT-5′
25bp	5′-ATTTCTTAATATTATCCTAAGCAAG-3′<br>3′-CTAAAGAATTATAATAGGATTCGTT-5′
26bp	5′-ATTTCTTAATATTATCCTAAGCAAGG-3′<br>3′-CTAAAGAATTATAATAGGATTCGTTC-5′
28bp	5′-AATTTCTTAATATTATCCTAAGCAAGGG-3′<br>3′-CTTAAAGAATTATAATAGGATTCGTTCC-5′

PmrA-binding sites are colored in red (half-1) and blue (half-2)

Supplementary Table 2. RMSDs of the three PmrA-DNA complex structures.

RMSD (Å)	Complex-1	Complex-2	Complex-3	
Complex-1		1.76	0.90	All residues included[a]
Complex-2	0.84		2.24	
Complex-3	0.64	1.08		
	PmrA-2 DBD excluded[b]			

[a] Cα of Residue 1-219 in both PmrA-1 and PmrA-2 were used to calculate RMSD.

[b] Cα of PmrA-1 residue 1-219 and PmrA-2 residue 1-121 were used in calculation.

Supplementary Table 3. H-bond networks in the REC-DBD interfaces of 3 PmrA-DNA complex structures.

	REC	Distances (Å)			DBD
		**Complex-1**	**Complex-2**	**Complex-3**	
1	Met1[N]	3.38	3.70	2.42	Asp177[O]
2	Lys2[Nζ]	2.78	4.01	2.75	Asn176[Oδ1]
3	Lys2[Nζ]	2.86	3.24	3.55	Tyr179[O]
4	Gly24[O]	2.64	2.77[a]	3.41	Arg139[Nη1]
5	Cys27[N]	3.68	3.71[b]	2.64	Ser180[Oγ]
6	Asn43[Nδ2]	3.69	6.15	2.95	Asn176[Oδ1]
7	Asn43[Nδ2]	3.57	6.23	3.90	Glu184[Oε2]
8	His44[N]	2.69	3.34	3.75	Asn176[Oδ1]
9	Ser46[Oγ]	3.23	3.43	3.34	Arg160[Nη2]
10	Asn120[Oδ1]	3.08	3.58	3.68	Arg160[Nη1]
11	Asn120[Oδ1]	3.91	H_2O[c]	3.12	Lys164[Nζ]

[a] The distance of the H-bond interaction is between Glu23[O] and Arg139[Nη1].

[b] The distance of the H-bond interaction is between Cys27[O] and Ser180[Oγ].

[c] The H-bond interaction between 2 residues is mediated by a water molecule.

Supplementary Table 4. Exchange parameters obtained from fitting ^{13}C-^{1}H multiple-quantum CPMG relaxation dispersion data for methyl groups of PmrA in the presence or absence of DNA to a two-site exchange model.

Residue	R_{ex} (s^{-1})[a]	p_B (%)	k_{ex} (s^{-1})	$\Delta\omega_C$ (ppm)	$\Delta\omega_H$ (ppm)	χ^2/DF[b]
With promoter DNA						
V26 γ2	14.15	8.2 ± 9.0	534 ± 146	0.38 ± 0.11	0.001 ± 0.001	2.1
L63 δ1	8.05	11.9 ± 3.8	255 ± 100	2.80 ± 0.22	0.646 ± 0.013	1.1
I127 δ1	6.18	9.2 ± 1.4	493 ± 78	0.28 ± 0.03	0.062 ± 0.004	1.1
L134 δ2	17.91	4.9 ± 2.3	662 ± 484	0.99 ± 0.22	0.086 ± 0.036	2.8
V136 γ1	11.60	11.1 ± 3.5	593 ± 121	0.53 ± 0.11	0.040 ± 0.021	1.8
V136 γ2	15.21	14.5 ± 4.4	379 ± 108	0.43 ± 0.06	0.073 ± 0.006	2.3
L140 δ2	8.85	12.6 ± 8.1	473 ± 188	0.31 ± 0.12	0.063 ± 0.017	0.8
L158 δ1	23.47	7.0 ± 5.1	643 ± 294	0.97 ± 0.28	0.087 ± 0.036	2.1
I173 δ1	8.05	7.3 ± 2.6	854 ± 126	0.35 ± 0.07	0.055 ± 0.015	1.3
L174 δ2	7.65	9.2 ± 1.9	506 ± 165	1.74 ± 0.27	0.179 ± 0.021	3.4
L216 δ1	9.20	8.6 ± 4.5	571 ± 161	0.35 ± 0.10	0.065 ± 0.017	1.7
L216 δ2	17.05	10.4 ± 2.4	637 ± 104	0.44 ± 0.06	0.053 ± 0.004	2.6
Without promoter DNA						
L40 δ1	3.01	2.0 ± 2.4	172 ± 98	0.62 ± 0.12	0.030 ± 0.019	5.2
L143 δ1	4.36	2.9 ± 2.6	112 ± 81	1.44 ± 0.09	0.001 ± 0.001	3.9
I194 δ1	3.81	5.7 ± 3.1	731 ± 81	0.28 ± 0.19	0.079 ± 0.026	2.3

[a] R_{ex} value was calculated using the equation $R_{ex} = R_{2,eff}(50Hz) - R_{2,eff}(1000Hz)$ from 850-MHz CPMG relaxation dispersion data

[b] χ^2/DF describes the quality of the fit. DF is the degree of freedom and was calculated by DF = (number of experimental data points) – (number of parameters) – 1.
