# Supplementary figures and images for: Structure and dynamics of polymyxin-resistance-associated response regulator PmrA in complex with promoter DNA
Source: Nat Commun. 2015 Nov 13;6:8838. doi: 10.1038/ncomms9838 (PMC4660055; doi:10.1038/ncomms9838)

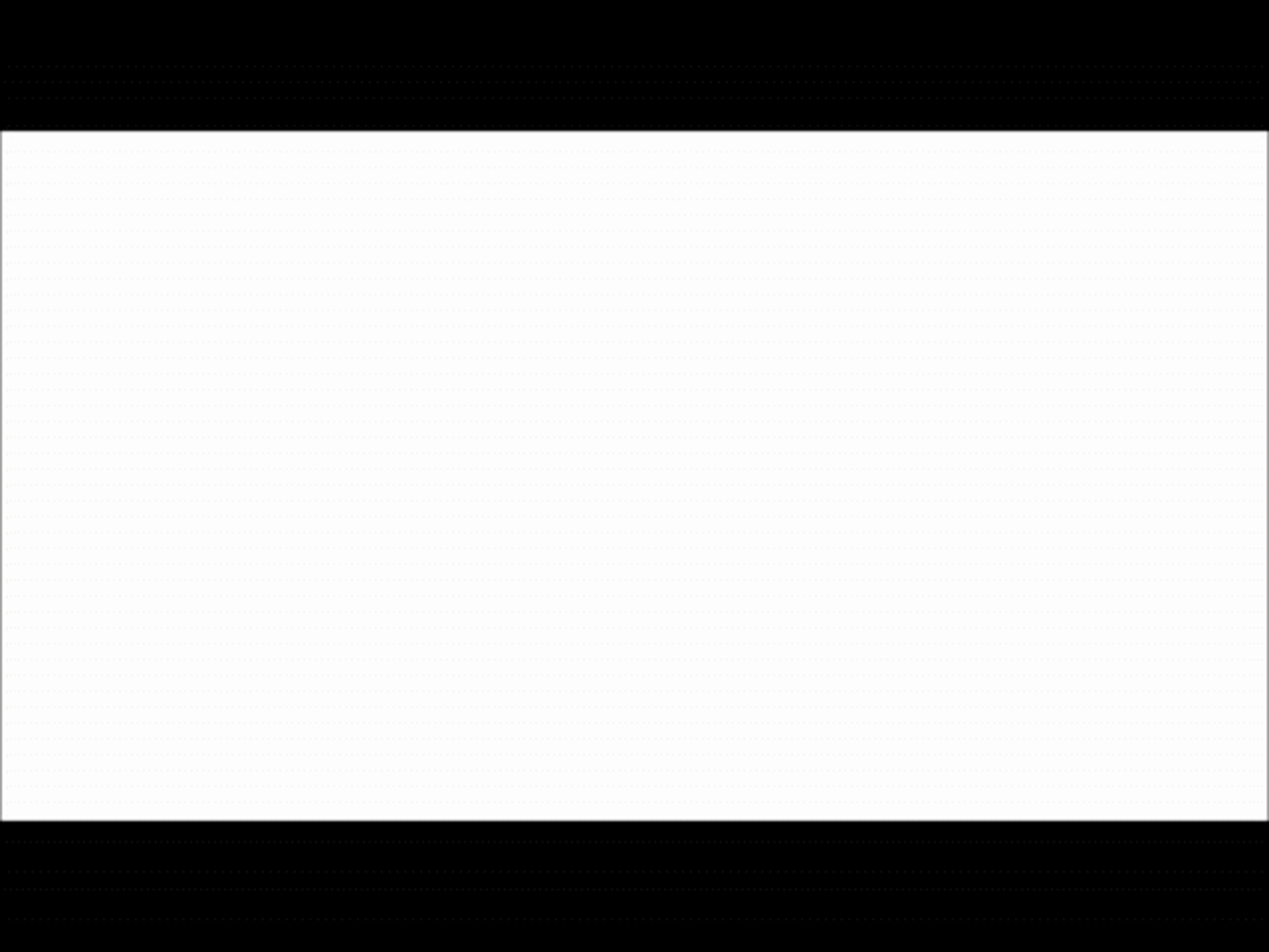

Supplement: Supplementary Movie 1 — The REC-DBD interdomain dynamics of PmrA. When the PmrA-DNA complex (PmrA-1, PmrA-2 and DNA are in green, yellow and blue, respectively) is recognized by the σ4 domain of RNAPH, greater REC-DBD interdomain dynamics allow the REC dimer to rotate more freely to adopt a suitable orientation that can best contact with the RNAPH to form the initial closed promoter complex for transcription initiation. [file ncomms9838-s2.tif]
